# Comparative efficacy of digital health interventions for depression and anxiety symptoms in adolescents and young adults: a systematic review and bayesian network meta-analysis

**DOI:** 10.1186/s13034-026-01042-3

**Published:** 2026-02-14

**Authors:** Haozhe Wang, Jiayi Yao, Shiguan Jia, Wenjia Chen, Yujie Guan, Jiahao Liu, Mingyu Liao, Enliang Hu, Qinghua Zhang

**Affiliations:** 1 School of Physical Education, China University of Mining and Technology, Xuzhou, 221116 China; 2 The Second Clinical College, Heilongjiang University of Chinese Medicine, Harbin, Heilongjiang China; 3 Department of Physical Training, Institute of Aviation Safety and Security, China Civil Aviation Flight Academy, Chengdu, China; 4 Shanghai, China

**Keywords:** Adolescents, Young adults, Digital health interventions, Depression, Anxiety, Network meta-analysis

## Abstract

**Background:**

Depression and anxiety symptoms in adolescents and young adults represent a significant global public health challenge. Digital health interventions (DHIs) offer potential solutions to supplement traditional mental health services, though the relative efficacy of different types of interventions remains unclear.

**Objective:**

This study aims to systematically compare the treatment effects of digital health interventions driven by different mechanisms on depression and anxiety symptoms in this population through a Bayesian network meta-analysis.

**Methods:**

A systematic search was conducted in major databases such as PubMed, Embase, and PsycINFO (up to September 2025), including randomized controlled trials (RCTs) targeting depression or anxiety symptoms in individuals aged 12–25 years. Interventions were categorized based on treatment mechanisms into four types: cognitive behavioral therapy-based digital interventions (CBT-DI), third-wave digital therapies (TWDT), general digital mental health support (GDMHS), and technology-enhanced innovative interventions (TEII). The primary outcome measure was the standardized mean difference (SMD), with the cumulative ranking probability assessed using the surface under the cumulative ranking curve (SUCRA).

**Results:**

A total of 18 RCTs involving 5, 821 participants were included. Network meta-analysis indicated that CBT-DI achieved the highest surface under the cumulative ranking curve (SUCRA) values for both depression (79.3%) and anxiety (83.4%). In pairwise comparisons with no intervention controls, CBT-DI demonstrated a statistically significant improvement in anxiety symptoms (SMD = 0.33, 95% CrI: 0.05 to 0.69). However, for depression, the improvement associated with CBT-DI did not reach statistical significance (SMD = 0.44, 95% CrI: -0.02 to 0.91), suggesting that the high ranking probability reflects a potential trend rather than confirmatory evidence of superiority. TWDT and GDMHS demonstrated moderate efficacy for both symptoms, ranking above usual care and no intervention controls. The evidence quality assessment (GRADE) indicated that the primary outcomes were of low to moderate quality.

**Conclusion:**

Digital health interventions, particularly CBT-based interventions (CBT-DI), were associated with statistically significant improvements in anxiety symptoms. For depression, while CBT-DI ranked highest in probability, it did not demonstrate statistical superiority over controls. Given the imprecision in effect estimates, CBT-DI may be considered a potential complementary measure within a stepped-care mental health system. Results should be interpreted with caution due to wide credible intervals, and further high-quality studies are required to confirm these findings.

**Supplementary Information:**

The online version contains supplementary material available at 10.1186/s13034-026-01042-3.

## Introduction

Adolescence and Young Adulthood (AYAs) represent a critical window in individual psychological development, marked by significant physiological changes, shifts in social roles, and neural-cognitive reorganization [1]. According to the World Health Organization (WHO) and neuroscience consensus, this phase spans from approximately ages 10 to 24, characterized by the ongoing development of the prefrontal cortex and dynamic integration of emotional regulation circuits [2–4]. Given that the maturation of the prefrontal cortex and emotional regulation is a biological process that continues into the mid-20s, studying the 12–25 age group as a whole aligns with the continuous nature of neurodevelopment and more accurately reflects the practical need for a smooth transition from adolescent to adult mental health services [5–6]. WHO data indicates that anxiety disorders affect 4.1% of adolescents aged 10–14 and 5.3% of those aged 15–19 globally [7]. In the 18–24 age group, the prevalence of depression is even higher, with U.S. data showing that approximately 15.8% of individuals experience major depressive episodes [8]. More concerningly, the National Comorbidity Survey-Adolescent (NCS-A) reports that as many as 49.5% of adolescents will experience some form of mental health disorder during their lifetime [9]. However, due to resource limitations and factors such as lack of awareness, a significant proportion of this affected population is unable to access timely psychological support.

Digital Health Interventions (DHIs) have emerged as a potential means to improve the accessibility of mental health services [10], addressing the resource gaps left by traditional approaches. While face-to-face psychological therapies, such as cognitive behavioral therapy (CBT), have demonstrated moderate to large effect sizes, they face challenges such as geographical limitations and high costs [11]. DHIs encompass a range of technological forms, including web platforms, mobile applications, and virtual reality, which offer private and immediate support, closely aligning with the usage habits of the “digital native” generation [12]. Previous evidence suggests that digital CBT-based interventions show positive trends in alleviating symptoms [13]. However, DHIs present considerable complexity and heterogeneity in clinical practice, and their intervention effects and comparability across studies may be influenced by multiple key dimensions. These include the theoretical orientation distinguishing classic CBT from third-wave therapies, levels of guidance (e.g., guided vs. purely self-help), platform formats (including apps, web platforms, and gamified designs), intervention intensity, and follow-up time points [14].

Despite the rapid growth of related research, the current body of evidence exhibits a fragmented characteristic. The vast majority of studies focus solely on comparisons between a single type of digital tool and no intervention control, with a lack of direct “head-to-head” comparative evidence between different intervention approaches or mechanisms [15]. Due to the scattered nature of direct comparison evidence, the high heterogeneity in intervention content and delivery methods, and inconsistent definitions of control conditions, traditional pairwise meta-analyses struggle to accurately assess the relative efficacy of different strategies within a unified statistical framework. Therefore, Bayesian network meta-analysis (NMA) becomes particularly necessary to integrate both direct and indirect evidence. Additionally, in order to overcome the limitations posed by technological iteration and to provide guidance for clinical decision-making, this study defines the network nodes based on treatment theory orientation, supplemented by classifications of delivery formats. The aim is to identify the core drivers that support behavioral change in this population.

Therefore, this study seeks to systematically evaluate the comparative efficacy of different mechanisms-driven digital health interventions for depression and anxiety symptoms in adolescents and young adults (12–25 years old) through Bayesian network meta-analysis (NMA). By integrating both direct and indirect evidence, the study will probabilistically rank interventions based on various psychological principles, including classic CBT, third-wave therapies, general support, and technology-enhanced interventions. Considering the continuity of neurodevelopment during the AYA stage, this approach aims to provide high-quality evidence for precise mental health services for this population.

## Methods

### Protocol and registration

This study has been registered with PROSPERO (CRD420251157120). We followed the Preferred Reporting Items for Systematic Reviews and Meta-Analyses (PRISMA) [16] extension for network meta-analysis [17].

### Search strategy

This study searched PubMed, Embase, Cochrane Central Register of Controlled Trials, Web of Science, and Scopus databases from inception to September 15, 2025. Relevant studies were supplemented by manually searching references of included literature and relevant systematic reviews. The detailed search strategy is provided in Supplementary Appendix 1. We employed the following MeSH terms and keywords: “Mobile Applications”, “Video Games”, “Therapy, Computer-Assisted”, “Virtual Reality”, “Artificial Intelligence”, “Depression”, “Anxiety”, “adolescent”, and “clinical trials as topic”.

### Literature selection

Two researchers independently managed and screened the literature using EndNote X9 [18]. After removing duplicates, the titles, abstracts, and full texts were reviewed according to the inclusion and exclusion criteria. Any disagreements during the screening process were resolved through discussion or adjudicated by a third researcher.

Inclusion criteria: (1) Study design: Randomized controlled trials (RCTs); (2) Study population: Adolescents and young adults (12–25 years old) diagnosed or screened with depression/anxiety symptoms [19]; (3) Intervention: Digital technology-based psychological interventions (e.g., CBT-DI, virtual reality, mobile applications, etc.); (4) Control: Usual care, no intervention control (e.g., waitlist), or other active control; (5) Outcomes and data: Depression or anxiety symptoms assessed using standardized scales, with complete data (e.g., means and standard deviations) available for calculating effect sizes.

Exclusion criteria: (1) Non-randomized controlled trial designs (e.g., cohort studies, prospective studies, case-control studies, cross-sectional studies, etc.); (2) Studies without full texts available; (3) Studies that did not report mental health outcomes or used non-standardized assessment tools; (4) Studies where baseline differences between the intervention and control groups were significant; (5) Conference abstracts, reviews, editorials, letters, or other publications without original data; (6) Insufficient data to calculate effect sizes, and unable to obtain data even after contacting the authors.

### Data extraction

Data extraction was performed independently by two researchers and cross-checked, with any disagreements resolved through discussion. In addition to extracting basic study characteristics and intervention details, we specifically focused on extracting continuous data for the primary outcomes immediately following the intervention. To ensure the accuracy of effect size estimation and effectively handle attrition bias, we strictly used the actual sample size for analysis rather than the baseline randomized sample size. Variance indicators were carefully reviewed, and any data reporting only standard errors (SE) were converted to standard deviations (SD) using the formula: ${\mathrm{SD}}\,{\text{ = }}\,{\mathrm{SE}}\, \times \,\sqrt {\mathrm{N}}$ [18]. The data extraction followed the principle of prioritizing post-intervention means and standard deviations; when these were unavailable or baseline imbalance occurred, baseline-to-endpoint change scores were extracted, with missing standard deviations estimated following the Cochrane Handbook [20].

This study classifies digital mental health interventions using a mechanism-based approach. Although the digital interventions included in the study differ in dimensions such as “level of guidance” (guided vs. self-help) and “technology delivery mode” (app vs. web platform), recent evidence suggests that the therapeutic theoretical mechanism, rather than technical features or guidance format alone, better predicts the clinical effectiveness heterogeneity of digital interventions [21]. Therefore, to ensure theoretical homogeneity of the network nodes, we did not use the level of guidance as a primary classification criterion. Instead, we followed the NIHR classification framework and best practices for network meta-analysis [22], categorizing interventions into four mutually exclusive categories based on core therapeutic theories: (1) Cognitive Behavioral Therapy-based Digital Interventions (CBT-DI), Including all digital interventions using core CBT techniques, such as cognitive restructuring and behavioral activation. (2) Third-Wave Digital Therapies (TWDT), Integrating intervention methods based on acceptance, mindfulness, and value-oriented approaches (e.g., ACT, MBCT). (3) General Digital Mental Health Support (GDMHS): Interventions that provide only psychological education, self-monitoring, or general support, representing non-specific therapeutic factors. (4) Technology-Enhanced Innovative Interventions (TEII): Emerging technological applications primarily driven by virtual reality, AI chatbots, or gamification mechanisms [23]. In terms of control group classification, to address the heterogeneity introduced by different control conditions, we strictly followed the typology standards for mobile health trials proposed by Goldberg et al. We clearly distinguish two independent control nodes: (1) No Intervention Control (NIC), Including only waitlist or no treatment conditions, serving as a baseline for evaluating absolute efficacy. (2) Treatment as Usual (TU): Including standard care from schools or communities or routine medical services, serving as an active control for evaluating relative efficacy. For groups marked as “active control” in the original studies but actually including specific digital intervention components (e.g., simple attention control apps), these were reclassified into the appropriate intervention node (e.g., GDMHS). This classification scheme minimizes within-node heterogeneity while ensuring network connectivity and statistical power.

### Risk of bias assessment

The quality of finally included literature was assessed using the Cochrane Collaboration Risk of Bias tool (RoB 2.0) [24], encompassing five bias risk dimensions: bias in the randomization process, bias due to deviations from intended interventions, bias due to missing outcome data, bias in measurement of outcomes, and bias in selection of reported results. Each dimension was classified as low risk, some concerns, or high risk according to Cochrane standards. Literature quality was assessed separately by two researchers, with disagreements resolved by a third researcher.

### Data synthesis and analysis

This study used R software (version 4.5.0) with the gemtc package to perform Bayesian NMA, requiring pre-installation of JAGS software (version 4.3.1). Network plots were constructed using the igraph package, with node size representing sample size and line thickness representing the number of studies, using a star layout centered on the intervention with the largest sample size.

For continuous outcomes, SMD and 95% CrI were calculated, employing a random-effects model (likelihood = “normal”, link = “identity”, linearModel = “random”). An inconsistency model (UME) was first run to test network inconsistency, followed by a consistency model for primary analysis, comparing Deviance Information Criterion (DIC) values to evaluate model fit. When networks formed closed loops, the node-splitting method was used to test local inconsistency.

Bayesian Markov Chain Monte Carlo (MCMC) settings were: 4 chains (n.chain = 4), adaptation period of 20, 000 iterations (n.adapt = 20000), formal iterations of 50, 000 (n.iter = 50000), and thinning parameter of 1. Gelman-Rubin diagnostics (PSRF < 1.1) were used to assess convergence, with trace plots and density plots drawn to monitor convergence status.

SUCRA values (0-100%) were used to rank interventions, with higher values indicating better efficacy. The probability of each intervention achieving each ranking position was calculated, with results displayed through cumulative ranking probability plots (stacked bar charts) and individual ranking probability plots (grouped bar charts). The mtc.anohe function was used to calculate I² to assess heterogeneity (I² > 50% suggests substantial heterogeneity).

The primary results were presented using a relative effect table (league table) to display pairwise comparisons between interventions. Sensitivity analyses were conducted using a leave-one-out approach. Publication bias was assessed using Egger’s linear regression test. Finally, the certainty of the body of evidence was graded according to the GRADE framework. All analyses were performed in R version 4.5.0 (full analytical procedures are provided in Appendix 2), and the analysis code and datasets will be made publicly available upon publication.

## Results

### Study selection

**Fig. 1:**
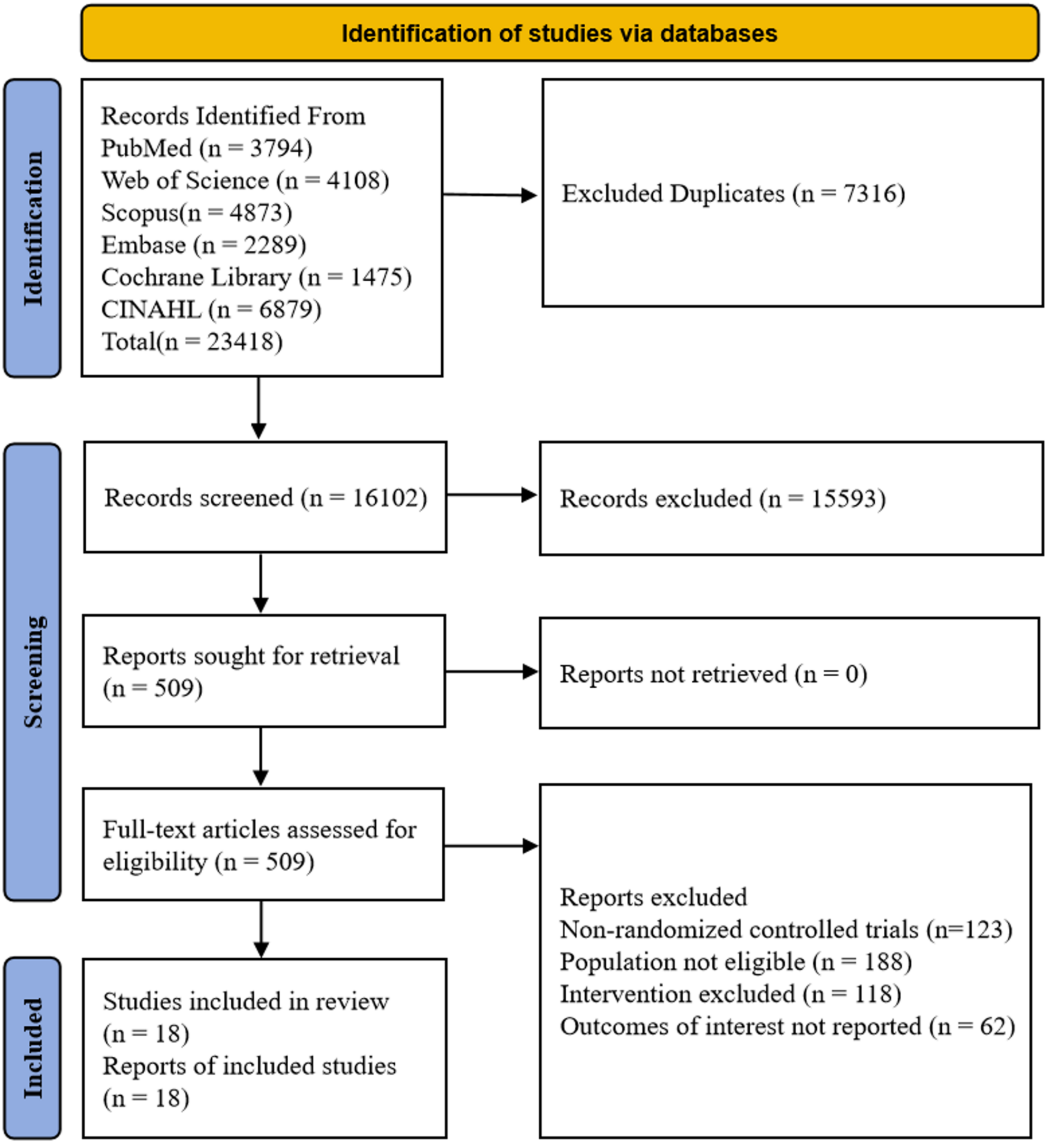
PRISMA 2020 flow diagram showing the process of study identification, screening, and inclusion. Adapted from Page et al. [16] (PRISMA 2020 statement).

A total of 23, 418 records were initially identified. After removing duplicates, 16, 102 records remained. Following title and abstract screening, 15, 593 records were excluded. The remaining 509 reports were reviewed in full text. A total of 491 reports were excluded due to issues related to study design (*n* = 123), population (*n* = 188), intervention (*n* = 118), or outcome measures (*n* = 62). Ultimately, 18 studies were included in the review. A detailed selection process is shown in Fig. 1.

This study ultimately included 18 randomized controlled trials, involving 5, 821 participants (see Table 1) [25–42]. Participants were aged between 12 and 25 years, covering the adolescent to young adult population. The studies were primarily conducted in high-income countries, with the highest number of studies conducted in Australia (5 studies) [27, 28, 30, 35, 39], followed by the Netherlands (3 studies) [32, 34, 41], China (2 studies) [31, 42], and the United States (2 studies) [26, 37]. The United Kingdom [25], Germany [29], Iran [33], Japan [36], and New Zealand [38] each had 1 study, with 1 multinational study involving the UK, Germany, Spain, and Belgium [40].

Based on therapeutic mechanisms, the digital interventions included in the study were widely distributed. Cognitive Behavioral Therapy-based Digital Interventions (CBT-DI) were the most commonly applied intervention type, with 12 studies [25, 27, 28, 29, 33, 36–42], mainly delivering structured cognitive restructuring and behavioral activation training through online modules or applications. Third-Wave Digital Therapies (TWDT) were involved in 3 studies [32, 34, 35], focusing on acceptance, mindfulness, and value-oriented psychological interventions. Technology-Enhanced Innovative Interventions (TEII) were also included in 3 studies [26, 31, 40], integrating emerging technologies such as AI chatbots, virtual reality, and motion-sensing games. General Digital Mental Health Support (GDMHS) was the main intervention in 2 studies [26, 30] and was widely used as an active control in 6 other studies [25, 27, 31, 37, 38, 40], primarily providing non-specific psychological education and support. Regarding control conditions and outcome assessments, there was some heterogeneity in study design. Nine studies used no intervention controls (e.g., waitlist) [26, 28, 29, 33–36, 41], 3 studies used usual care as a reference [30, 39, 42], and 6 studies used active controls for head-to-head comparisons [25, 27, 31, 37, 38, 40] to evaluate the relative effectiveness of the interventions. The outcome measures were diverse, with the majority of studies (17 studies) assessing depressive symptoms [25, 27–42] and 13 studies reporting anxiety symptoms [25– 26, 28–30, 32, 33, 35, 37, 39–42], using standardized scales such as PHQ-9 and GAD-7. Additionally, some studies also measured secondary outcomes such as quality of life, functional improvement, and treatment adherence, providing data to comprehensively assess the clinical value of digital interventions.

**Table 1:** Characteristics of included studies

Study	Country	Participants (intervention/control)		Interventions			Comparisongroup (s)	Depression	Anxiety	Other outcomes
		*n*	Age	Type	Sessions (times) week	Duration (w)				
Barr et al. (2017)	UK	45/46	12–18 years	CBT-DI	8 sessions total	1	GDMHS	BDI, MFQ	SCAS	QoL, functioning
Cioffi et al. [26]	USA	14/14/14	14–18 years	TEII, GDMHS	5/week	3	NIC	–	PASF	Stress, cognition
Deady et al. [27]	Australia	60/44	18–25 years	CBT-DI	4 sessions total	4	GDMHS	PHQ-9	–	Alcohol use
Farrer et al. [28]	Australis	244/243	18–25 years	CBT-DI	12 modules	6	NIC	PHQ-9	GAD-7	Distress, QoL
Funk et al. [29]	Germany	122/122	16–22 years	CBT-DI	Self-paced	6	NIC	IDS	GADQ-IV	RNT measures
Hallford et al. [30]	Australia	246/113	15–25 years	GDMHS	7 modules self-paced	24	TU	DASS-21	GAD-7	Rumination et al.
He et al. [31]	China	49/49	17–21 years	TEII	7 modules/1 per day	7	GDMHS	PHQ-9	–	Working alliance, usability
Hoek et al. [32]	Netherlands	22/23	12–21 years	TWDT	one lesson a week	5	NIC	CES-D	HADS-A	Client satisfaction
Javadi et al. [33]	Iran	20/20	Adolescents	CBT-DI	12 sessions, 2 days/week	8	NIC	DASS-21	DASS-21	Stress, psychological flexibility
Kramer et al. [34]	Netherlands	131/132	19.5 ± 1.7 years	TWDT	Mean 1.36 sessions (max 5)	9	NIC	CES-D	–	–
Manicavasagar et al. [35]	Australia	120/115	12–18 years	TWDT	Website use for 6 consecutive weeks	6	NIC	DASS-21	DASS-21	SWEMWBS (well-being)
Nagamitsu et al. [36]	Japan	69/72	13–18 years	CBT-DI	WCV (2 visits) + 2-week CBT app	24	NIC	DSRS-C	–	AHP-SF, RSES, PedsQL
Peake et al. [37]	USA	63/58	16.8 ± 2.5 years	CBT-DI	5 modules, self-paced (rec. 1/week)	5	GDMHS	PHQ-8	GAD-7	–
Stasiak et al. [38]	New Zealand	17/17	13–18 years	CBT-DI	7 modules (“The Journey”)	4–10 weeks	GDMHS	CDRS-R	–	RADS-2, PedsQL, ACS
Teesson et al. [39]	Australia	1015/1150	13–14 years	CBT-DI	Online modules	104	TU	PHQ-8	GAD-7	Substance use knowledge, alcohol use
Watkins et al. [40]	UK/Germany/Spain/Belgium	178 (TEII) / 191 (CBT-DI) / 199 (GDMHS)	16–22 years	TEII, CBT-DI	–	48	GDMHS	PHQ-9	GAD-7	WEMWBS, WSAS, EQ-5D-3 L
Zanden et al. [41]	Netherlands	121/123	16–25 years	CBT-DI	1 sessions, weekly	6	NIC	CES-D	HADS-A	Mastery Scale, functioning
Zhou et al. [42]	China	275/265	Grades 10–11 (mean 16.8 years)	CBT-DI	11 sessions, weekly	11	TU	DASS-21 depression	DASS-21 anxiety	PSS-10 (stress), CISS-SFC (coping), ORS-4 (wellbeing)

Detailed descriptions of intervention protocols for each category are provided in Supplementary Appendix 3

CBT-DI: Cognitive Behavioral Therapy-based digital interventions; TWDT: Third-Wave Digital Therapies; GDMHS: General Digital Mental Health Support; TEII: Technology-Enhanced Innovative Interventions; NIC: No Intervention Control; TU: Treatment as Usual

### Risk of bias assessment

**Fig. 2:**
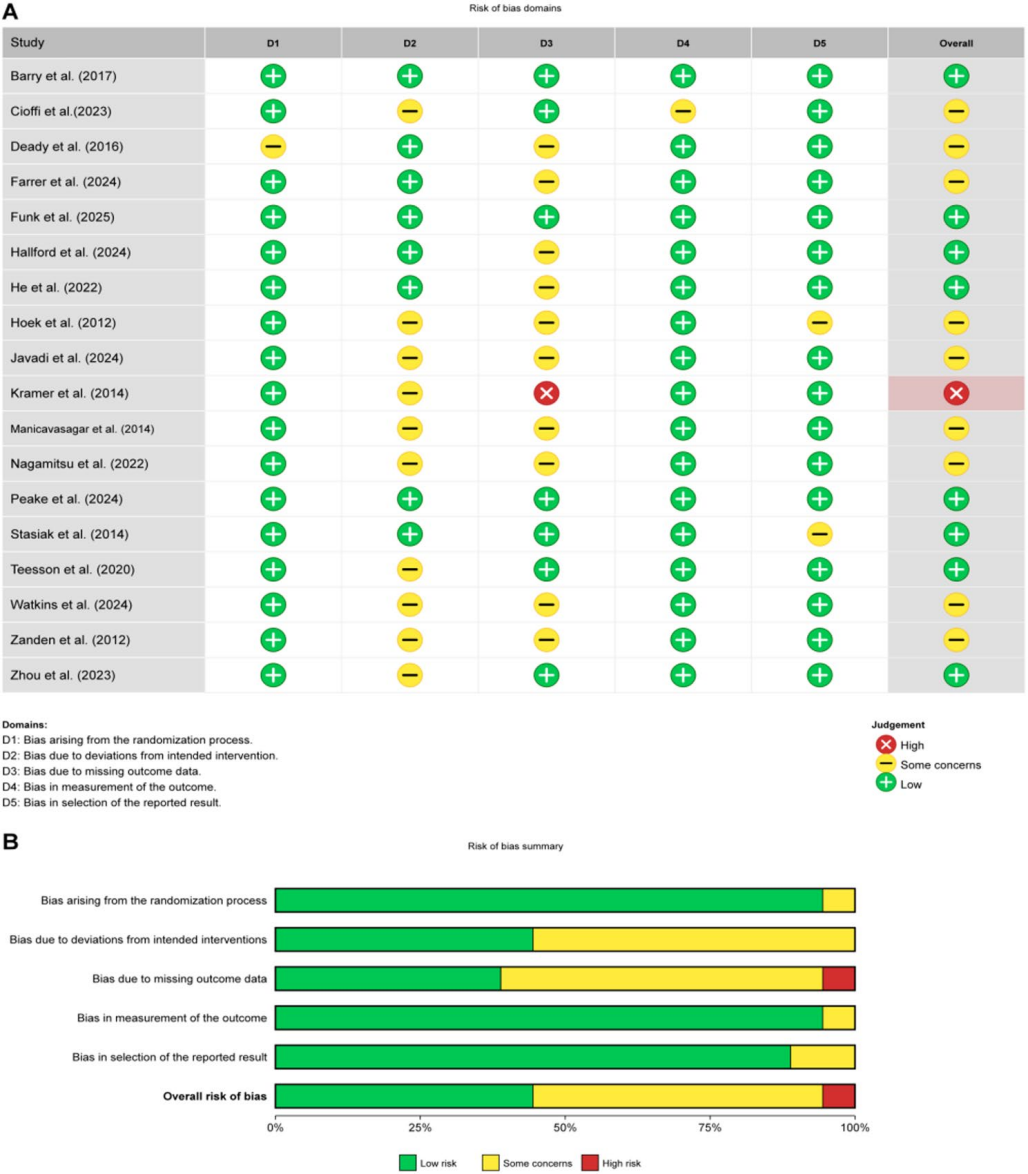
**A** Risk of bias summary: review of the authors judgments about each risk of bias item for each included study. **B** Risk of bias graph: review authors’ judgments about each risk of bias item, presented as percentage of included studies

The overall methodological quality of the 18 included studies was considered acceptable (see Fig. 2). During the randomization process, the vast majority of studies (94.4%, 17/18) performed well, with only 1 study [27] having an uncertain risk. Due to the nature of digital mental health interventions, which make double-blinding difficult to implement, the risk of deviations from the intended intervention dimensions was relatively high, with 55.6% (10/18) of the studies having an uncertain risk in this area. Missing outcome data were a major source of bias, with 55.6% (10/18) of studies presenting an uncertain risk due to insufficient reporting of methods for handling missing data. Only 1 study [34] was rated as high risk due to high attrition and lack of appropriate statistical remedies.

Thanks to the use of standardized scales and adherence to pre-registration protocols, the quality of outcome measurement and selective reporting dimensions was high, with low-risk percentages of 94.4% and 88.9%, respectively.

The overall risk of bias assessment showed that 8 studies (44.4%) were at low risk, 9 studies (50.0%) had uncertain risk, and only 1 study (5.6%) was rated as high risk. Despite limitations in blinding and data completeness in some studies, the overall quality of the included studies was sufficient to support subsequent analyses.

### Network meta-analysis

#### Depression outcome network analysis results

**Fig. 3:**
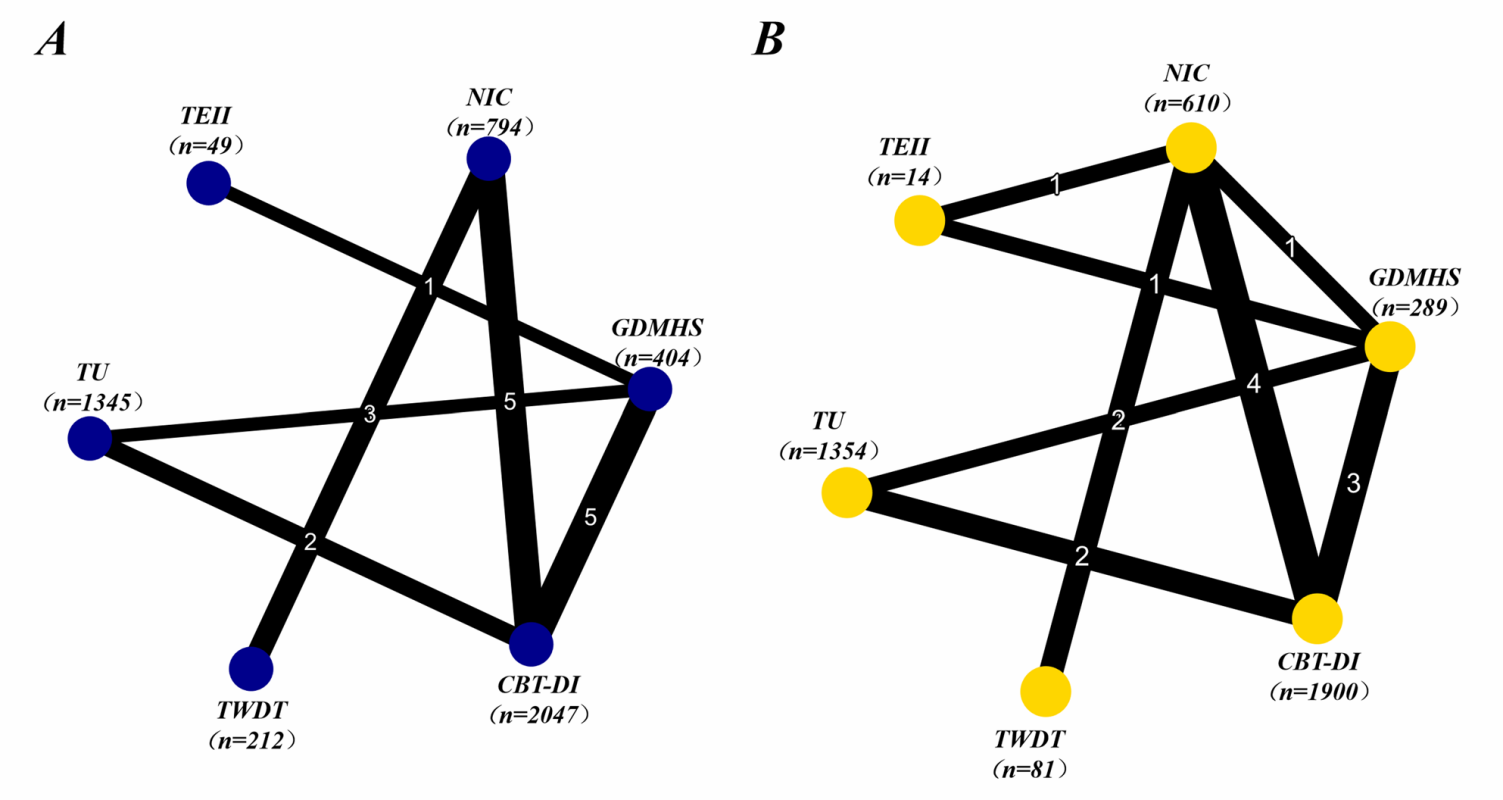
Network plot for (**A**) depression outcomes and (**B**) anxiety outcomes. CBT-DI: Cognitive Behavioral Therapy-based digital interventions; GDMHS: General Digital Mental Health Support; NIC: No Intervention Control; TEII: Technology-Enhanced Innovative Interventions; TU: Treatment as Usual; TWDT: Third-Wave Digital Therapies

This study included 18 randomized controlled trials on depressive outcomes, involving 5, 821 participants. The network diagram shows good connectivity between the intervention nodes, with the CBT-DI node having the most direct comparison evidence (Fig. 3A).

The fit of the Bayesian consistency model was similar to that of the inconsistency model (DIC difference = 0.24), and all parameters converged well (PSRF = 1.0). The overall network heterogeneity was high (Global I² = 92%). The node-splitting method revealed no significant local inconsistency (all *P* > 0.05), indicating statistical consistency between direct and indirect evidence. The high heterogeneity likely reflects the clinical diversity of the included studies, including participant characteristics (age range 12–25 years), a wide variety of measurement tools, and the different forms of digital intervention implementation (e.g., levels of guidance, interaction modes).

Pairwise comparisons based on the league table (Table 2) show that, compared to no intervention control (NIC), CBT-DI was the ranked highest in SUCRA intervention (SMD = 0.44, 95% CrI: -0.02 to 0.91). Although the 95% CrI spans the null value of 0, its point estimate is in the moderate effect range, and it ranks highly in the SUCRA ranking. Other interventions, such as TEII (SMD = 0.48) and TWDT (SMD = 0.37), also showed trends of being showed a favorable trend to the control group, but with wider confidence intervals and greater uncertainty.

**Fig. 4:**
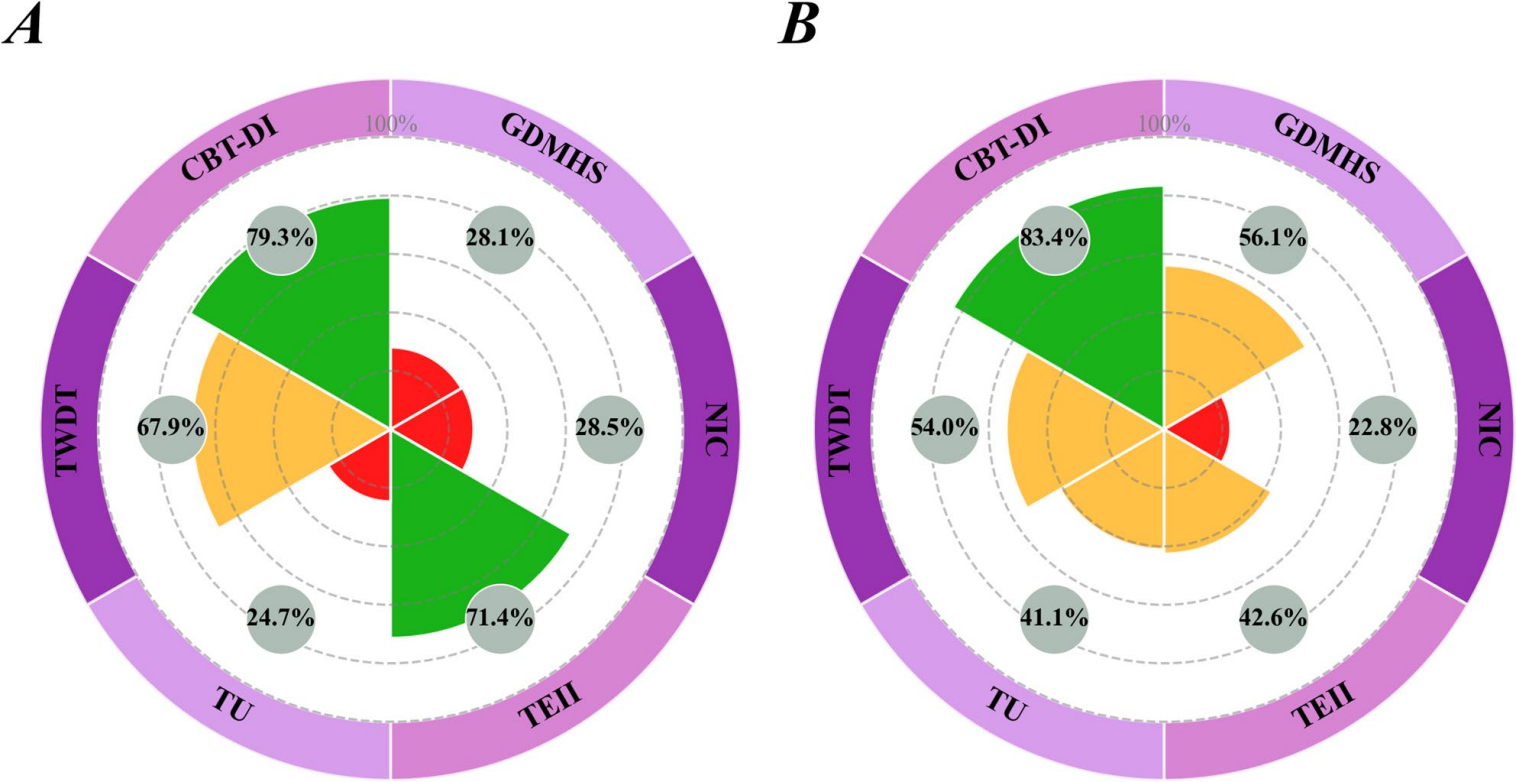
SUCRA rankings for (**A**) depression outcomes and (**B**) anxiety. The Vitruvian plot displays the surface under the cumulative ranking (SUCRA) values [43], ranging from 0 to 100%. Colour coding: green represents better performance, yellow represents moderate performance, red/orange represents poorer performance. CBT-DI: Cognitive Behavioral Therapy-based digital interventions; GDMHS: General Digital Mental Health Support; NIC: No Intervention Control; TEII: Technology-Enhanced Innovative Interventions; TU: Treatment as Usual; TWDT: Third-Wave Digital Therapies

The SUCRA cumulative ranking probability plot (Fig. 4A) shows that CBT-DI has the highest likelihood of being the best intervention (SUCRA = 79.3%), followed by TEII (71.4%) and TWDT (67.9%). Although TEII and TWDT rank higher overall, their wider confidence intervals, combined with relative efficacy results, suggest that the certainty of evidence is lower than for CBT-DI. NIC (28.5%), GDMHS (28.1%), and TU (24.7%) ranked the lowest.

**Table 2:** League table for depression outcomes

	CBT-DI	GDMHS	NIC	TEII	TU	TWDT
CBT-DI	–	0.45 (0.02, 0.92)	0.44 (– 0.02, 0.91)	– 0.04 (– 1.17, 1.12)	0.5 (– 0.06, 1.15)	0.07 (– 0.68, 0.85)
GDMHS	– 0.45 (– 0.92, – 0.02)	–	– 0.01 (– 0.67, 0.61)	– 0.5 (– 1.55, 0.55)	0.05 (– 0.6, 0.73)	– 0.38(– 1.28, 0.49)
NIC	– 0.44 (– 0.91, 0.02)	0.01 (– 0.61, 0.67)	–	– 0.48 (– 1.7, 0.77)	0.06 (– 0.66, 0.85)	– 0.37 (– 0.97, 0.24)
TEII	0.04 (– 1.12, 1.17)	0.5 (– 0.55, 1.55)	0.48 (– 0.77, 1.7)	–	0.55 (– 0.68, 1.81)	0.11 (– 1.27, 1.48)
TU	– 0.5 (– 1.15, 0.06)	– 0.05 (– 0.73, 0.6)	– 0.06 (– 0.85, 0.66)	– 0.55 (– 1.81, 0.68)	–	– 0.43 (– 1.43, 0.51)
TWDT	– 0.07(– 0.85, 0.68)	0.38 (– 0.49, 1.28)	0.37 (– 0.24, 0.97)	– 0.11 (– 1.48, 1.27)	0.43 (– 0.51, 1.43)	–

Positive values indicate greater symptom improvement for row intervention compared to column intervention; bold numbers indicate 95% credible intervals not including 0 (statistically significant)

CBT-DI: Cognitive Behavioral Therapy-based digital interventions; TWDT: Third-Wave Digital Therapies; GDMHS: General Digital Mental Health Support; TEII: Technology-Enhanced Innovative Interventions; NIC: No Intervention Control; TU: Treatment as Usual

#### Anxiety outcome network analysis results

A total of 17 RCTs (involving 5, 877 participants) were included for anxiety outcomes. The network diagram shows that all interventions were connected through direct or indirect comparisons (Fig. 3B). The model convergence was good, with all parameters having a PSRF of exactly 1.00, and the DIC difference between the consistency and inconsistency models was less than 5, confirming the applicability of the consistency assumption.

In the pairwise comparisons of relative efficacy (Table 3), only CBT-DI showed statistically significant improvement compared to no intervention control (NIC), with a standardized mean difference (SMD) of 0.33 (95% CrI: 0.05 to 0.69). Other digital interventions (including TEII, TWDT, and GDMHS) also showed trends of improvement compared to NIC, but their 95% CrI spanned the null value of 0. No significant statistical differences were found between the active interventions.

The SUCRA-based ranking results further revealed the cumulative probability advantages of different interventions (Fig. 4B). CBT-DI ranked the highest (SUCRA = 83.4%), followed by GDMHS (56.1%), TWDT (54.0%), TEII (42.6%), and Treatment as Usual (TU) (41.1%), with NIC (22.8%) performing the worst.

Global heterogeneity analysis showed relatively stable results (I² = 68.16%), and no significant local inconsistency was found through the node-splitting method (all *P* > 0.05). This result confirms that the derived effect of anxiety improvement based on the current evidence chain is statistically reliable.

**Table 3:** League table for anxiety outcomes

	CBT-DI	GDMHS	NIC	TEII	TU	TWDT
CBT-DI	–	0.14 (– 0.19, 0.49)	0.33 (0.05, 0.69)	0.26 (– 0.58, 1.12)	0.22 (– 0.14, 0.62)	0.16 (– 0.41, 0.81)
GDMHS	– 0.14 (– 0.49, 0.19)	–	0.19 (– 0.22, 0.66)	0.11 (– 0.73, 0.97)	0.08 (– 0.36, 0.54)	0.01 (– 0.63, 0.73)
NIC	– 0.33 (– 0.69, – 0.05)	– 0.19 (– 0.66, 0.22)	–	– 0.08 (– 0.93, 0.75)	– 0.1 (– 0.62, 0.35)	– 0.17 (– 0.68, 0.35)
TEII	– 0.26 (1.12, 0.58)	– 0.11 (– 0.97, 0.73)	0.08 (– 0.75, 0.93)	–	– 0.03 (– 0.95, 0.88)	– 0.09 (– 1.07, 0.91)
TU	– 0.22 (– 0.62, 0.14)	– 0.08 (– 0.54, 0.36)	0.1 (– 0.35, 0.62)	0.03 (– 0.88, 0.95)	–	– 0.07 (– 0.75, 0.68)
TWDT	– 0.16 (– 0.81, 0.41)	– 0.01 (– 0.73, 0.63)	0.17 (– 0.35, 0.68)	0.09 (– 0.91, 1.07)	0.07 (– 0.68, 0.75)	–

Positive values indicate greater symptom improvement for row intervention compared to column intervention; bold numbers indicate 95% credible intervals not including 0 (statistically significant)

CBT-DI: Cognitive Behavioral Therapy-based digital interventions; TWDT: Third-Wave Digital Therapies; GDMHS: General Digital Mental Health Support; TEII: Technology-Enhanced Innovative Interventions; NIC: No Intervention Control; TU: Treatment as Usual

#### Sensitivity analysis

To verify the robustness of the Bayesian network meta-analysis results, a sensitivity analysis was conducted using the leave-one-out approach. By sequentially removing each study and re-estimating the combined effect size, this method tests whether the results are dominated by individual extreme data or small sample studies.

The analysis results (Appendix 4) show that the primary outcome measures exhibited high robustness. For depressive symptoms, despite differences in study sample sizes, the overall combined effect size remained stable at around − 0.425 after removing any single study, with no reversal in direction or significant changes in statistical significance. For anxiety symptoms, the summary effect size fluctuated between − 0.18 and − 0.23 after removing individual studies, further confirming the reliability of the effect on anxiety symptom improvement, with no undue influence from any single study.

#### Publication bias

Egger’s linear regression test and the Trim and Fill method were used to comprehensively assess potential publication bias and small-study effects (Appendix 4). For depressive outcomes, although Egger’s test indicated funnel plot asymmetry (*P* = 0.0012), the Trim and Fill analysis did not identify any missing studies, confirming that the original effect size estimate was not substantially distorted by potential bias. For anxiety outcomes, Egger’s test did not reveal significant publication bias (*P* = 0.233). Although the Trim and Fill method identified and corrected for two potentially missing studies, the corrected effect size direction remained consistent with the original results. Overall, multiple sensitivity analyses confirmed the robustness of the primary conclusions of this study.

#### Certainty of evidence assessment

According to the GRADE standards (Table 4), the overall quality of evidence in this study was rated as low to moderate. The certainty for anxiety outcomes was rated as moderate, downgraded primarily for imprecision because several comparisons among active interventions had wide credible intervals that included 0. The rating for depressive outcomes was low, further downgraded due to significant asymmetry indicated by Egger’s test (*P* = 0.0012), suggesting a potential risk of publication bias. Other domains (risk of bias, inconsistency, and indirectness) were all rated as low risk.

**Table 4:** GRADE evidence quality assessment

Outcome	No. of studies (participants)	Risk of bias	Inconsistency	Indirectness	Imprecision	Publication bias	Certainty
Depression	18 RCTs (*n* = 5821)	Not serious	Not serious	Not serious	Serious^a^	Serious^b^	Low ⊕⊕⊖⊖
Anxiety	17 RCTs (*n* = 5877)	Not serious	Not serious	Not serious	Serious^a^	Not serious	Moderate ⊕⊕⊕⊖

^a^Downgraded for Imprecision: 95% Credible Intervals cross the null value (0), indicating uncertainty in the effect estimate

^b^Downgraded for Publication Bias: Significant asymmetry in Egger’s test (P = 0.0012) suggests potential small-study effects or selective reporting

## Discussion

### Summary of main findings

This study systematically evaluated the relative efficacy of different digital health interventions (DHIs) in improving depressive and anxiety symptoms in adolescents and young adults aged 12 to 25 years, based on 18 randomized controlled trials. Unlike previous studies that focused on single interventions, this study integrated both direct and indirect evidence through network meta-analysis. The results showed that, compared to usual care or no intervention control, digital health interventions generally exhibited a favorable trend in improving psychological symptoms.

In the specific intervention strategy comparisons, Cognitive Behavioral Therapy-based Digital Interventions (CBT-DI) demonstrated a favorable pattern of results across both diagnostic categories. Based on the SUCRA probability ranking, CBT-DI ranked first in improving both depressive (SUCRA = 79.3%) and anxiety (SUCRA = 83.4%) symptoms. Notably, for anxiety symptoms, CBT-DI was the only active intervention whose 95% credible interval did not include the null value (0), providing statistical evidence of its efficacy.

However, results for depressive symptoms warrant cautious interpretation. Although CBT-DI ranked highest in the probability ranking, its pairwise comparison with no intervention control (SMD = 0.44, 95% CrI: -0.02 to 0.91) did not reach statistical significance. This suggests that the current evidence reflects a potential trend toward clinical benefit rather than conclusive evidence of efficacy. Moreover, although Third-Wave Digital Therapies (TWDT) and General Digital Mental Health Support (GDMHS) followed behind in the rankings, their wider credible intervals indicate that their efficacy estimates carry a degree of imprecision.In conclusion, the existing evidence suggests that CBT-DI may be considered a viable option in adolescent digital mental health services, but it also emphasizes the importance of accounting for statistical uncertainty when making clinical decisions.

### Possible explanations and comparison with literature

#### Efficacy for depressive symptoms

The results of the present network meta-analysis indicate that, for improving depressive symptoms in adolescents and young adults, Cognitive Behavioral Therapy–based Digital Interventions (CBT-DI) demonstrated the most favorable relative efficacy, ranking highest in the cumulative probability analysis (SUCRA = 79.3%). This ranking exceeded that of Third-Wave Digital Therapies (TWDT), General Digital Mental Health Support (GDMHS), and Technology-Enhanced Innovative Interventions (TEII). Although the pairwise comparison between CBT-DI and no intervention control (NIC) did not reach conventional statistical significance (SMD = 0.44, 95% CrI: −0.02 to 0.91), its high placement in the probabilistic ranking suggests a potential clinical value when considered as a prioritized intervention option.

This pattern is consistent with previous evidence on digital interventions for adolescent depression.A large-scale meta-analysis by Cuijpers et al., incorporating 409 trials, demonstrated that CBT-based digital interventions retain the core therapeutic components of traditional face-to-face CBT and are associated with moderate to large effect sizes in the treatment of depressive symptoms [11]. Similarly, a recent systematic review by Wu et al. supported the effectiveness of internet-based CBT for adolescent depression, highlighting that its standardized intervention structure not only contributes to the stability of treatment effects but may also enhance treatment adherence through gamified and interactive design features [44]. Although the present study observed relatively wide credible intervals, likely attributable to sample and intervention heterogeneity, the overall direction of effect aligns with this existing body of large-scale evidence, thereby reinforcing the potential role of CBT-DI within the spectrum of digital interventions for adolescent depression.

The relative advantage of CBT-DI in alleviating depressive symptoms may be explained by its clearly defined and theoretically grounded therapeutic mechanisms, which directly target core psychopathological processes underlying adolescent depression—namely maladaptive cognitive schemas and behavioral withdrawal.

First, CBT-DI incorporates digitalized cognitive restructuring modules that assist adolescents in identifying and challenging maladaptive thinking patterns, such as catastrophizing and dichotomous thinking. A component-level analysis of mainstream mental health applications by Wasil et al. indicated that interventions integrating cognitive restructuring and emotion-monitoring functions tend to demonstrate synergistic effects in reducing depressive symptoms [45]. By translating these core techniques into structured and interactive exercises, CBT-DI enables adolescents to repeatedly practice the identification and modification of automatic negative thoughts in daily life, thereby interrupting maladaptive cognitive–emotional cycles [46].

Second, behavioral activation represents another key active component of CBT-DI. Adolescents with depression often become trapped in a maladaptive cycle characterized by low mood, reduced activity, and diminished pleasure [47]. Through digitalized scheduling tools, activity tracking, and real-time feedback systems, CBT-DI encourages gradual engagement in activities that promote both mastery and pleasure [48]. Compared with non-specific general digital support (GDMHS), this structured intervention format may provide clearer behavioral scaffolding for adolescents whose cognitive control capacities are still developing, thereby facilitating greater treatment adherence and supporting symptom improvement [49].

Taken together, although the statistical uncertainty reflected by wide credible intervals warrants cautious interpretation of the magnitude of effect, the integration of probabilistic ranking results with prior mechanistic evidence suggests that CBT-DI remains one of the more promising digital intervention approaches for improving depressive symptoms in adolescents and young adults.

#### Efficacy for anxiety symptoms

In the intervention of anxiety symptoms, Cognitive Behavioral Therapy–based Digital Interventions (CBT-DI) similarly demonstrated considerable clinical applicability. According to the SUCRA-based probability ranking, CBT-DI ranked first (83.4%) and was the only active intervention in the present analysis that showed a statistically significant difference compared with no intervention control (NIC) (SMD = 0.33, 95% CrI: 0.05 to 0.69). This finding indicates that, in the treatment of anxiety symptoms among adolescents and young adults, CBT-DI provides a statistically significant benefit than natural symptom progression or waitlist conditions. Nevertheless, this result should be interpreted with appropriate caution. Although CBT-DI showed numerically greater effects than other digital interventions, such as Third-Wave Digital Therapies (TWDT) and General Digital Mental Health Support (GDMHS), direct comparisons between these active interventions did not reach statistical significance. This suggests that different categories of digital interventions may all confer a certain degree of therapeutic benefit, while CBT-DI currently demonstrates a relatively higher probability of being the preferred option within the existing evidence framework. These findings are consistent with, and complementary to, the current high-quality evidence base. A meta-analysis by Ebert et al. reported that internet-based CBT exhibited favorable effects compared with waitlist controls in the treatment of adolescent anxiety [50]. Similarly, a systematic review by Wickersham et al. documented the effectiveness of CBT-DI across multiple anxiety subtypes, including generalized anxiety disorder and social anxiety. Although prior studies by Vøllestad et al. and Spijkerman et al. emphasized the effectiveness of acceptance- and mindfulness-based interventions (TWDT) for anxiety disorders [51, 52], the network comparison results of the present study suggest that, for a broader adolescent anxiety population, structured CBT-DI may represent a more stable and generalizable intervention option.

The relative advantage of CBT-DI in alleviating anxiety symptoms may be attributable to the digital translation of its core therapeutic components, particularly exposure and cognitive restructuring [53, 54]. Adolescent anxiety disorders are commonly characterized by avoidance of fear-related stimuli and catastrophizing appraisals of perceived threats [55].

First, CBT-DI typically incorporates standardized graded exposure modules [56, 57]. Digital platforms can leverage multimedia scenarios or guided real-life practice to support adolescents in gradually confronting anxiety-provoking stimuli within a safe and controllable environment [58]. Pennant et al. suggested that such programmatic exposure exercises facilitate the process of habituation, thereby reducing excessive physiological and psychological arousal [59]. Compared with Third-Wave Digital Therapies (TWDT), which place greater emphasis on emotional acceptance, the exposure-based components of CBT-DI are designed to directly disrupt the anxiety–avoidance reinforcement cycle, a mechanism that may be particularly relevant during the early stages of symptom management [60].

Second, CBT-DI provides structured cognitive coping strategies that assist adolescents in reappraising perceived threat cues in their environment. Through interactive online exercises, adolescents learn to identify and challenge maladaptive beliefs, such as the expectation that anxiety will inevitably lead to an inability to cope [61]. For adolescents whose cognitive control capacities are still developing, this form of structured skills training offers a concrete and accessible set of coping tools.

Notably, CBT-DI ranked highest in the probabilistic analyses for both depressive and anxiety outcomes in the present study. This finding supports its potential value as a transdiagnostic intervention approach. Many CBT-DI programs adopt design principles similar to those of the Unified Protocol, targeting shared mechanisms underlying negative emotion regulation across disorders. Given the high prevalence of comorbidity between depression and anxiety in adolescent populations, intervention strategies capable of addressing both symptom domains concurrently may be particularly applicable within school-based mental health services and primary care settings.

### Clinical and policy implications

The findings of the present study suggest that digital health interventions may have practical applicability in the development of a stratified and transdiagnostic mental health service system for adolescents and young adults.

First, CBT-DI shows potential for addressing symptoms across conditions, although its implementation might benefit from a stratified approach. Given the uncertainty surrounding its effects on depressive symptoms, clinical recommendations should remain cautious. CBT-DI could be considered as an initial intervention option for anxiety symptoms, whereas for moderate to severe depression, it may be more appropriately positioned as a supplementary tool to routine care or as a supportive intervention during waiting periods, rather than as a replacement for standard face-to-face treatment.

Second, General Digital Mental Health Support (GDMHS) may serve as a “pre-preventive layer” within stepped-care models. Although its therapeutic effects are relatively modest, its high scalability makes it well suited for large-scale mental health literacy promotion and preliminary screening. A progressive resource allocation strategy—whereby GDMHS is first disseminated broadly and individuals who do not respond are subsequently referred to more specialized services—may help optimize public health efficiency in resource-limited settings.

Third, while digital interventions can substantially enhance accessibility to mental health support by lowering help-seeking barriers, their implementation should be accompanied by strengthened evidence-based oversight. Health policy and decision-making bodies are advised to emphasize robust assessment standards for digital mental health tools. Consideration might be given to interventions with clearly defined theoretical mechanisms and clinical validation, while their integration into public service systems should be explored under conditions that ensure safety and effectiveness.

### Strengths and limitations

The present study was conducted in adherence to established methodological standards. To our knowledge, this is the first study to apply Bayesian network meta-analysis within a unified, mechanism-based framework to compare digital interventions for depressive and anxiety symptoms in adolescents and young adults. By integrating direct and indirect evidence, this approach moves beyond traditional technology-driven classifications to explore core psychotherapeutic mechanisms. Furthermore, the study followed the PRISMA-NMA guidelines and applied the GRADE framework to evaluate the certainty of evidence, enhancing the transparency of the findings.

However, the interpretation of the current evidence is subject to several limitations. First and foremost, imprecision in effect estimates represents a primary constraint. For several key comparisons—particularly regarding depressive symptoms—the 95% credible intervals were wide and included the null value (0). This indicates that the high SUCRA rankings should be interpreted as a probabilistic trend rather than definitive statistical superiority. This uncertainty also led to the downgrading of the certainty of evidence for some outcomes. Second, potential publication bias was observed. Although the trim-and-fill method did not identify missing studies, the significant funnel plot asymmetry for depressive outcomes (Egger’s *P* = 0.0012) suggests the presence of small-study effects. Consequently, the reported treatment effects may be overestimated and warrant cautious interpretation. Finally, clinical heterogeneity across included studies limits the generalizability of the findings. Variations in control conditions (e.g., waitlist versus treatment as usual) and intervention duration, along with a general lack of long-term follow-up data, constrain conclusions regarding the durability of treatment effects.In addition, the reliance on self-reported outcome measures (e.g., PHQ-9, GAD-7) rather than clinician-administered interviews may introduce response bias. Furthermore, due to the nature of digital interventions, most included trials could not implement double-blinding for participants. Consequently, the potential influence of demand characteristics or digital placebo effects on the observed outcomes cannot be completely ruled out.

## Conclusion

The present study indicates that digital health interventions represent a beneficial adjunctive approach for alleviating depressive and anxiety symptoms in adolescents and young adults. The findings from the Bayesian network meta-analysis suggest that Cognitive Behavioral Therapy–based Digital Interventions (CBT-DI) demonstrate a favorable pattern of results across outcomes, ranking highest in cumulative probability for both depressive and anxiety symptoms.

However, these findings must be interpreted in light of statistical uncertainty. While CBT-DI showed statistically significant improvements for anxiety, the effect on depressive symptoms did not reach statistical significance compared to controls. Therefore, the high SUCRA ranking for depression reflects a potential trend rather than conclusive evidence of superiority.

Based on current evidence, CBT-DI may be considered a viable option within the spectrum of digital mental health interventions, particularly as a complementary measure in stepped-care models rather than a standalone substitute for conventional care. General Digital Mental Health Support (GDMHS) and Third-Wave Digital Therapies (TWDT) also exhibited favorable trends and may serve as alternative options to address diverse clinical needs. Future research should prioritize high-quality head-to-head randomized controlled trials and place greater emphasis on long-term follow-up to clarify the sustained real-world benefits of digital mental health interventions.

## Supplementary Information

Below is the link to the electronic supplementary material.

Supplementary Material 1.

## Data Availability

Data are available from the corresponding author.

## Abbreviations

AYAs: Adolescents and Young Adults
CBT-DI: Cognitive behavioral therapy-based digital interventions
CrI: Credible interval
DHIs: Digital health interventions
DIC: Deviance information criterion
GDMHS: General digital mental health support
GRADE: Grading of recommendations assessment, development and evaluation
MCMC: Markov chain Monte Carlo
NIC: No intervention control
NMA: Network meta-analysis
PRISMA: Preferred reporting items for systematic reviews and meta-analyses
RCTs: Randomized controlled trials
RoB 2: Revised Cochrane risk-of-bias tool for randomized trials
SD: Standard deviation
SMD: Standardized mean difference
SUCRA: Surface under the cumulative ranking
TEII: Technology-enhanced innovative interventions
TU: Treatment as usual
TWDT: Third-wave digital therapies
WHO: World Health Organization
