# Supplementary material for: Comparative efficacy of digital health interventions for depression and anxiety symptoms in adolescents and young adults: a systematic review and bayesian network meta-analysis
Source: Child Adolesc Psychiatry Ment Health. 2026 Feb 14;20:25. doi: 10.1186/s13034-026-01042-3 (PMC12918560; doi:10.1186/s13034-026-01042-3)
Supplement: Supplementary file 1 — Supplementary Material 1. [file 13034_2026_1042_MOESM1_ESM.docx]

**Comparative Efficacy of Digital Health Interventions for Depression and Anxiety Symptoms in Adolescents and Young Adults: A Systematic Review and Bayesian Network Meta-Analysis**

Appendix 1: Search Strategy (Page 1)

Appendix 2: Methodological Details (Page 2)

Appendix 3: Detailed descriptions of intervention protocols (Page 12)

Appendix 4: Sensitivity Analysis And Publication Bias (Page 14)

**Appendix 1: Search Strategy**

Search strategy in PubMed

| Step | Search strategy |
| --- | --- |
| #1 | Search (“Video Games”[Mesh]) OR ((((((Game, Video) OR (Games, Video)) OR (Video Game)) OR (Computer Game)) OR (Game, Computer)) OR (Games, Computer)) |
| #2 | Search (“Therapy, Computer-Assisted”[Mesh]) OR ((((((((((((((Therapy, Computer Assisted) OR (Computer-Assisted Therapy)) OR (Computer Assisted Therapy)) OR (Computer-Assisted Therapies)) OR (Therapies, Computer-Assisted)) OR (Protocol-Directed Therapy, Computer-Assisted)) OR (Protocol Directed Therapy, Computer Assisted)) OR (Computer-Assisted Protocol-Directed Therapy)) OR (Computer Assisted Protocol Directed Therapy)) OR (Computer-Assisted Protocol-Directed Therapies)) OR (Protocol-Directed Therapies, Computer-Assisted)) OR (Therapies, Computer-Assisted Protocol-Directed)) OR (Therapy, Computer-Assisted Protocol-Directed)) OR (Therapy, Computer Assisted Protocol Directed)) |
| #3 | Search (“Cell Phone”[Mesh]) OR (((((((((((((((((((((((Phone, Cell) OR (Phones, Cell)) OR (Cellular Phone)) OR (Phone, Cellular)) OR (Phones, Cellular)) OR (Telephone, Cellular)) OR (Cellular Telephone)) OR (Telephones, Cellular)) OR (Portable Cellular Phone)) OR (Cellular Phone, Portable)) OR (Cellular Phones, Portable)) OR (Transportable Cellular Phone)) OR (Cellular Phone, Transportable)) OR (Cellular Phones, Transportable)) OR (Mobile Phone)) OR (Phone, Mobile)) OR (Phones, Mobile)) OR (Mobile Telephone)) OR (Telephone, Mobile)) OR (Telephones, Mobile)) OR (Car Phone)) OR (Phone, Car)) OR (Phones, Car)) |
| #4 | Search (“Virtual Reality”[Mesh]) OR ((((((((((((Virtual Reality, Educational) OR (Reality, Virtual)) OR (Educational Virtual Reality)) OR (Educational Virtual Realities)) OR (Reality, Educational Virtual)) OR (Virtual Realities, Educational)) OR (Virtual Reality, Instructional)) OR (Instructional Virtual Realities)) OR (Instructional Virtual Reality)) OR (Realities, Instructional Virtual)) OR (Reality, Instructional Virtual)) OR (Virtual Realities, Instructional)) |
| #5 | Search (((((((((Virtual reality exposure therapy) OR (VR)) OR (Virtual)) OR (Augmented)) OR (Computer)) OR (Exergam)) OR (Interactive)) OR (Wii)) OR (Kinect)) OR (Xbox) |
| #6 | Search (“Artificial Intelligence”[Mesh]) OR ((((((((((((((((((Intelligence, Artificial) OR (Computational Intelligence)) OR (Intelligence, Computational)) OR (Machine Intelligence)) OR (Intelligence, Machine)) OR (Computer Reasoning)) OR (Reasoning, Computer)) OR (AI (Artificial Intelligence))) OR (Computer Vision System)) OR (System, Computer Vision)) OR (Systems, Computer Vision)) OR (Vision System, Computer)) OR (Vision Systems, Computer)) OR (Knowledge Acquisition (Computer))) OR (Acquisition, Knowledge (Computer))) OR (Knowledge Representation (Computer))) OR (Knowledge Representations (Computer))) OR (Representation, Knowledge (Computer))) |

**Appendix 2: Detailed Bayesian NMA Results and Model Diagnostics**

Part A: Results for Depression Outcomes

| 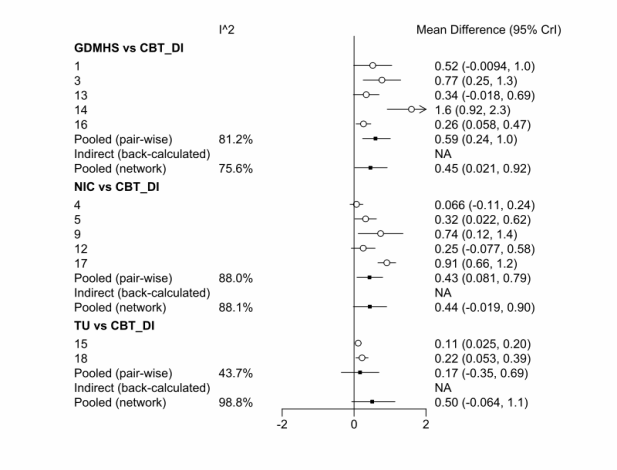 | 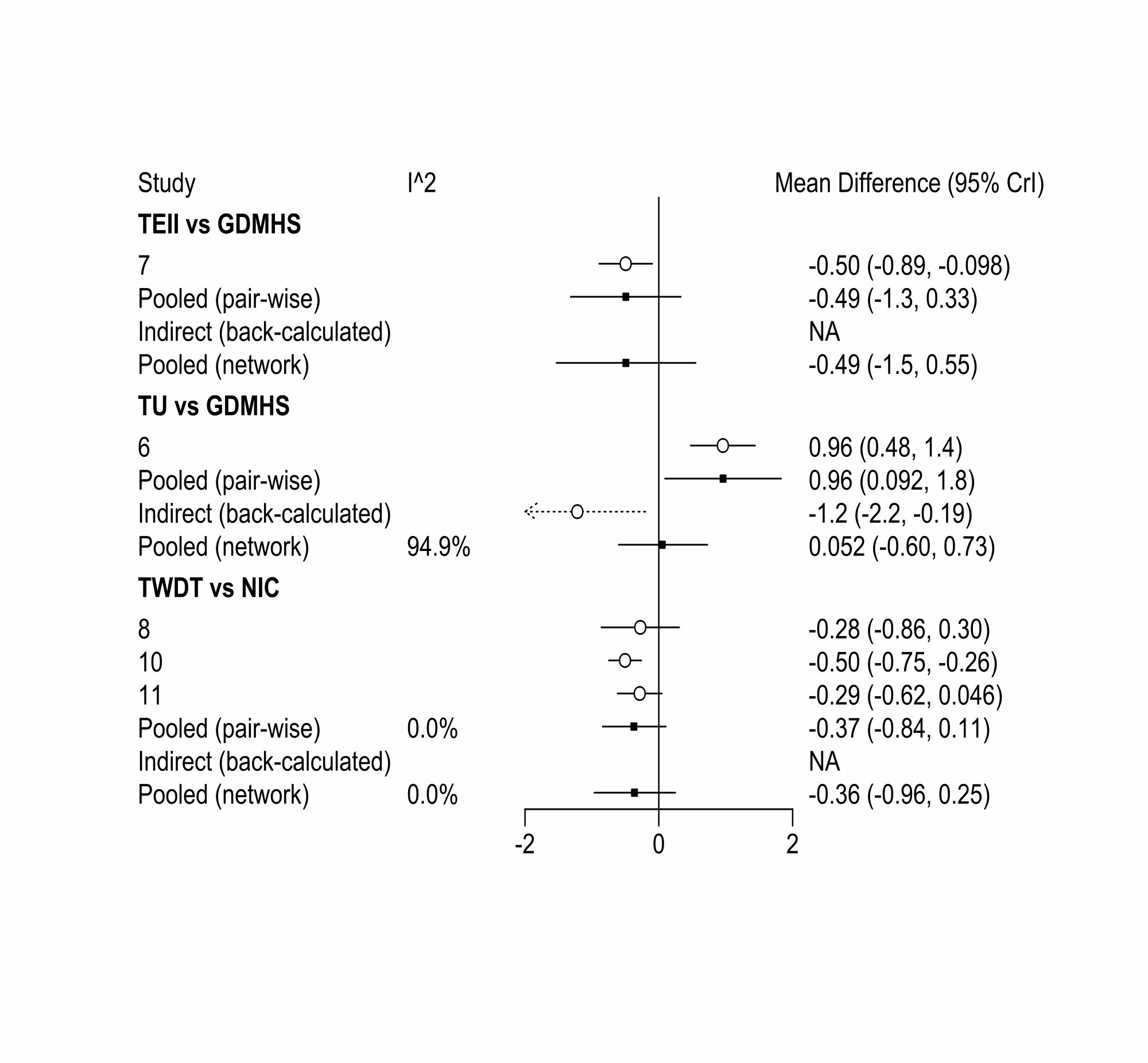 |
| --- | --- |

Figure S1. Detailed Forest Plots and Predictive Intervals (Depression).

Legend: This figure presents the forest plots summarizing the relative effect sizes (Standardized Mean Difference, SMD) for key pairwise comparisons in the depression network. The hollow circles represent the point estimates from individual studies, and the solid squares represent the pooled SMD from the network meta-analysis. The horizontal lines indicate the 95% Credible Intervals (CrI). If the horizontal line crosses the vertical line at 0, the difference is not statistically significant. The I2 value indicates the level of heterogeneity for each pairwise comparison.

| 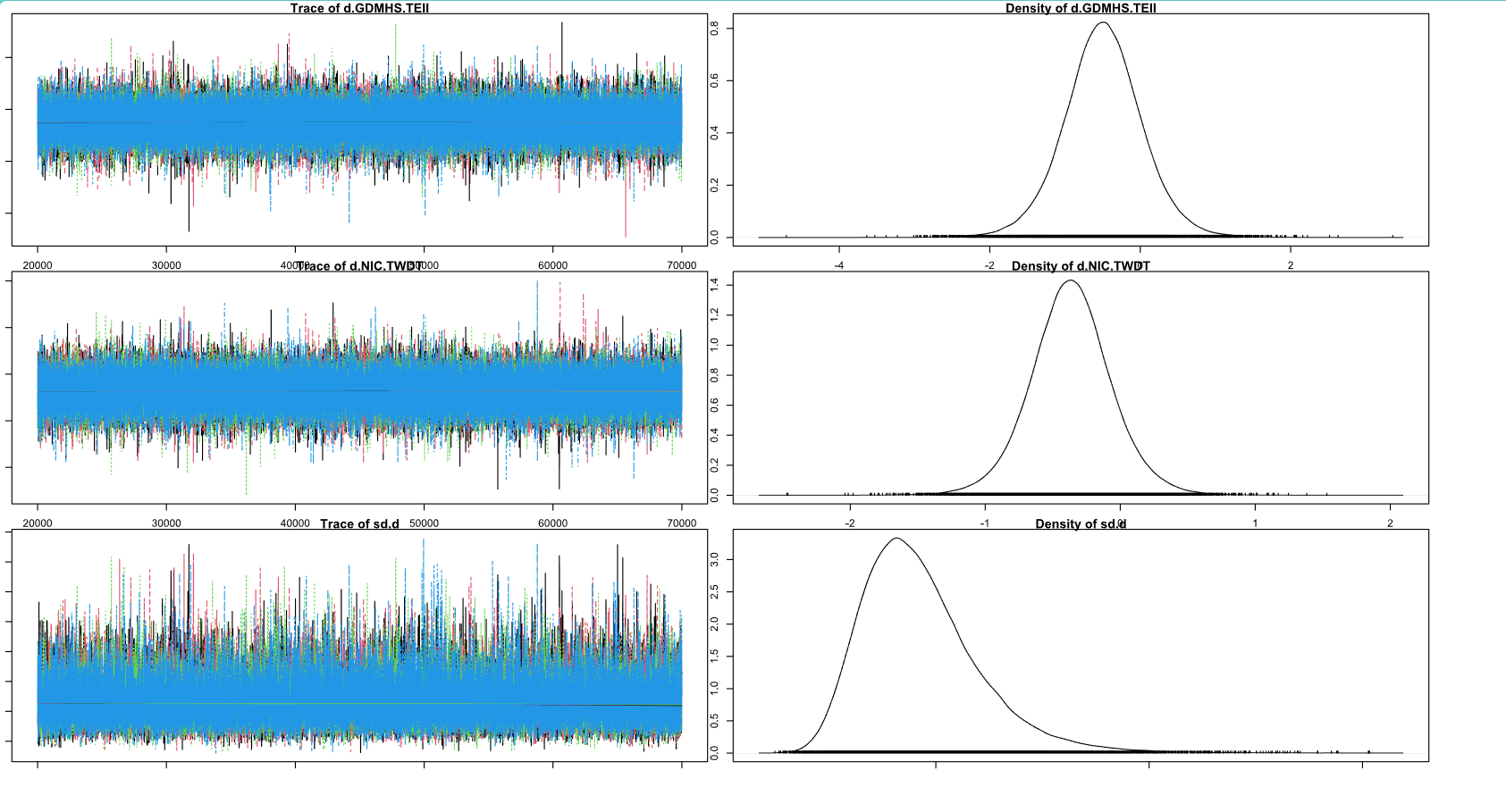 |
| --- |
| 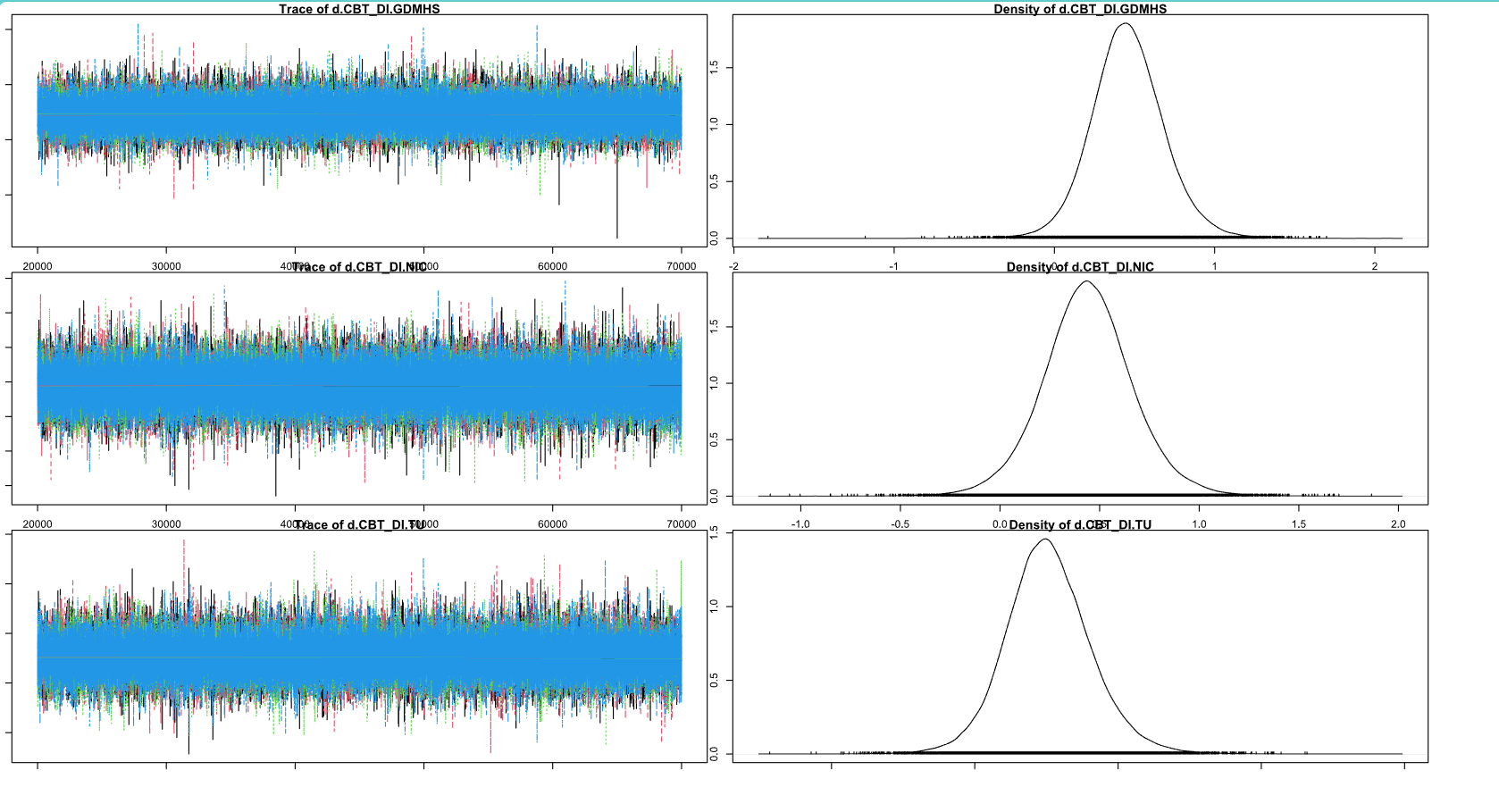 |
| 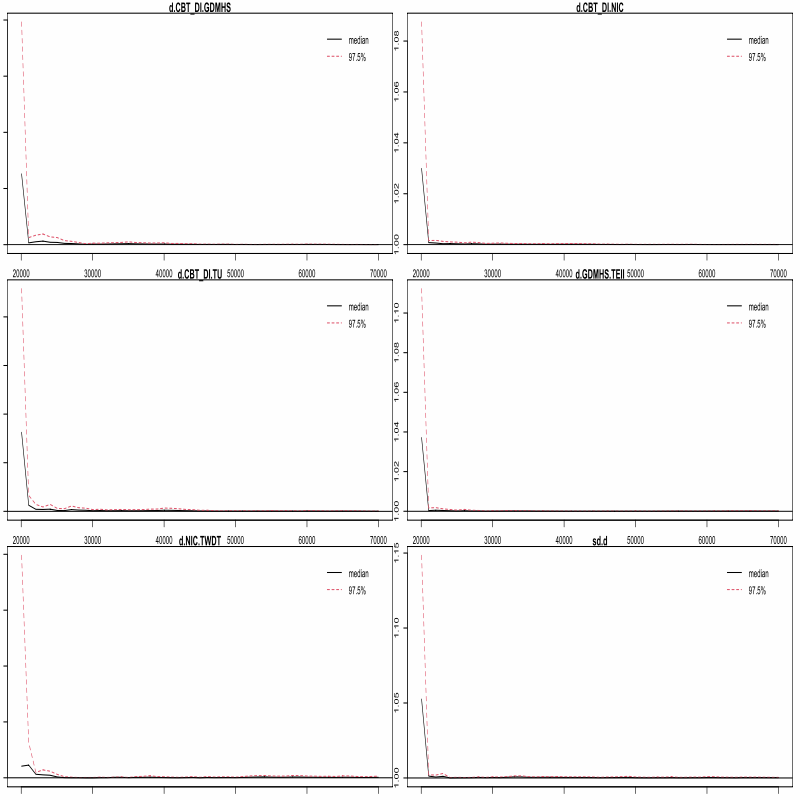 |
| 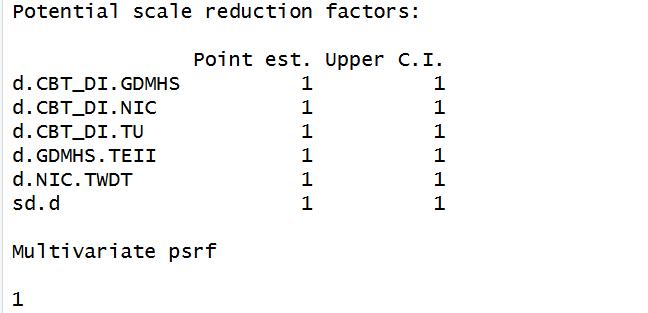 |

Figure S2. MCMC Convergence Diagnostics (Depression).

Legend: This composite figure displays the convergence diagnostics for the Bayesian model (Depression outcomes). (Trace Plots): The left panels show the sampling history of the MCMC chains. The overlapping of multiple chains (different colors) into a stable "caterpillar" shape indicates good mixing and convergence. (Density Plots): The right panels display the posterior density distributions of the estimated effect sizes, showing smooth, unimodal curves. (Gelman-Rubin Diagnostic): The bottom table presents the Potential Scale Reduction Factors (PSRF). All Point estimates are 1.0, confirming that the model has successfully converged.

| 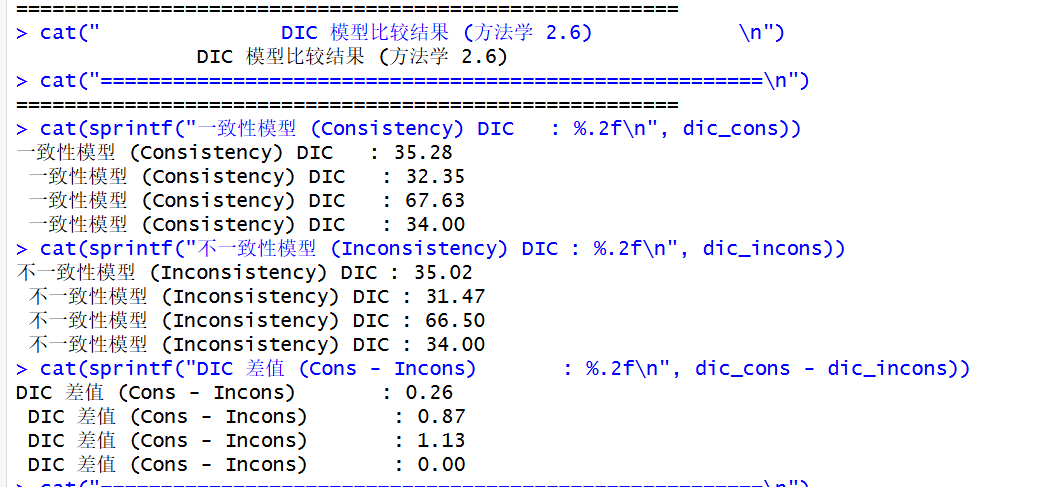 |
| --- |
| 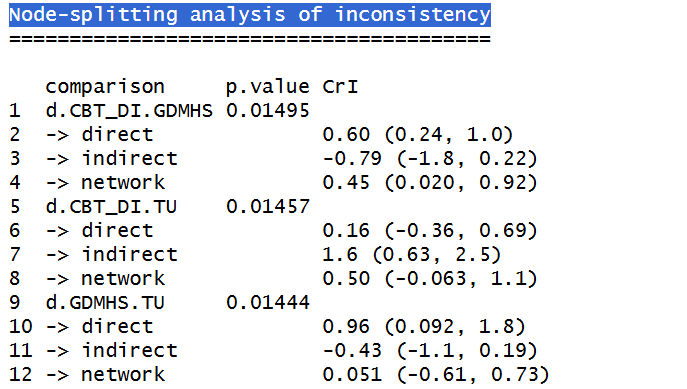 |

Figure S3. Inconsistency Assessment (Depression).

Legend: Node-splitting analysis was performed to assess local inconsistency between Direct Evidence (from head-to-head trials) and Indirect Evidence (inferred from the network). The table lists the SMD and 95% Credible Intervals (CrI) for each path. A P-value > 0.05 (e.g., P=0.79, P=0.85) indicates that there is no statistically significant difference between direct and indirect estimates, supporting the consistency assumption of the network.

Part B: Results for Anxiety Outcomes

| 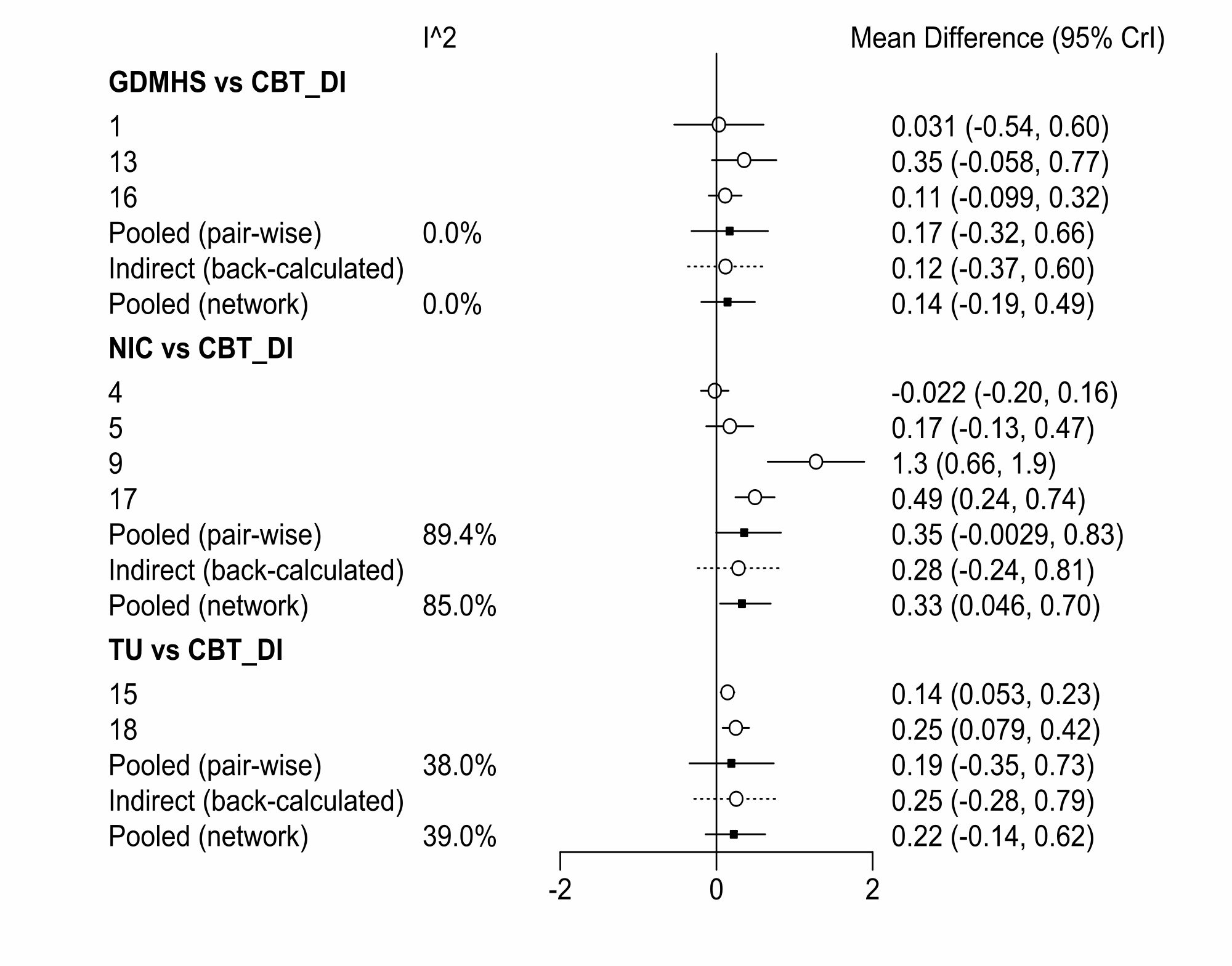 | 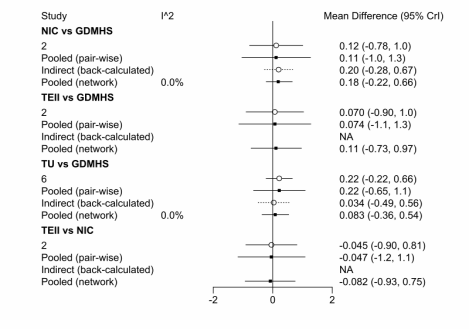 | 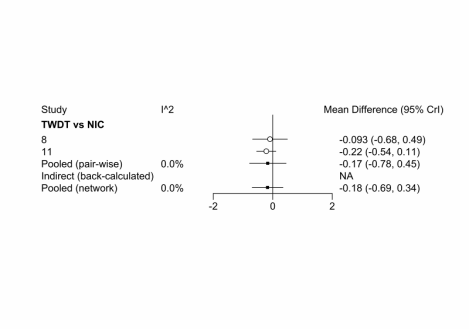 |
| --- | --- | --- |

Figure S4. Detailed Forest Plots and Predictive Intervals (Anxiety).

Legend: Forest plots summarizing the relative effect sizes (SMD) for pairwise comparisons in the anxiety network. The solid squares indicate the pooled effect estimates derived from the network meta-analysis, with horizontal lines representing the 95% Credible Intervals. The plots illustrate the magnitude and direction of effects for interventions (e.g., CBT-DI, GDMHS) compared to controls or other treatments. Comparisons where the interval does not cross zero are considered statistically significant.

| 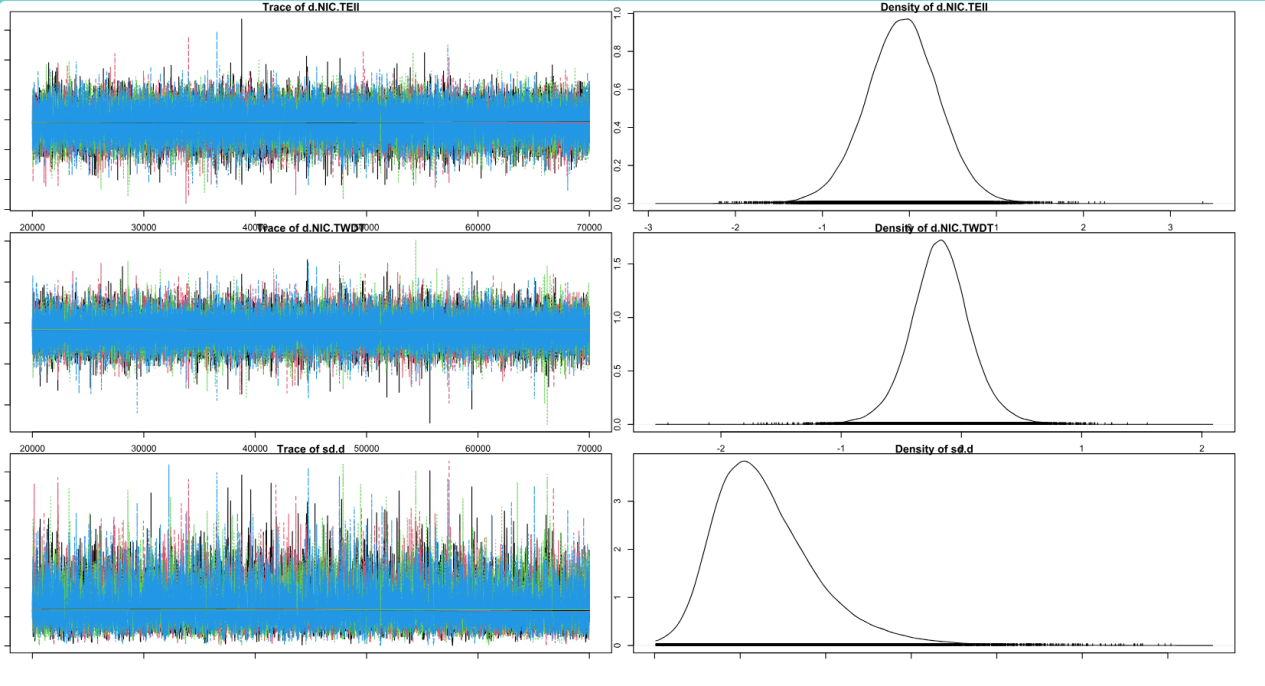 |
| --- |
| 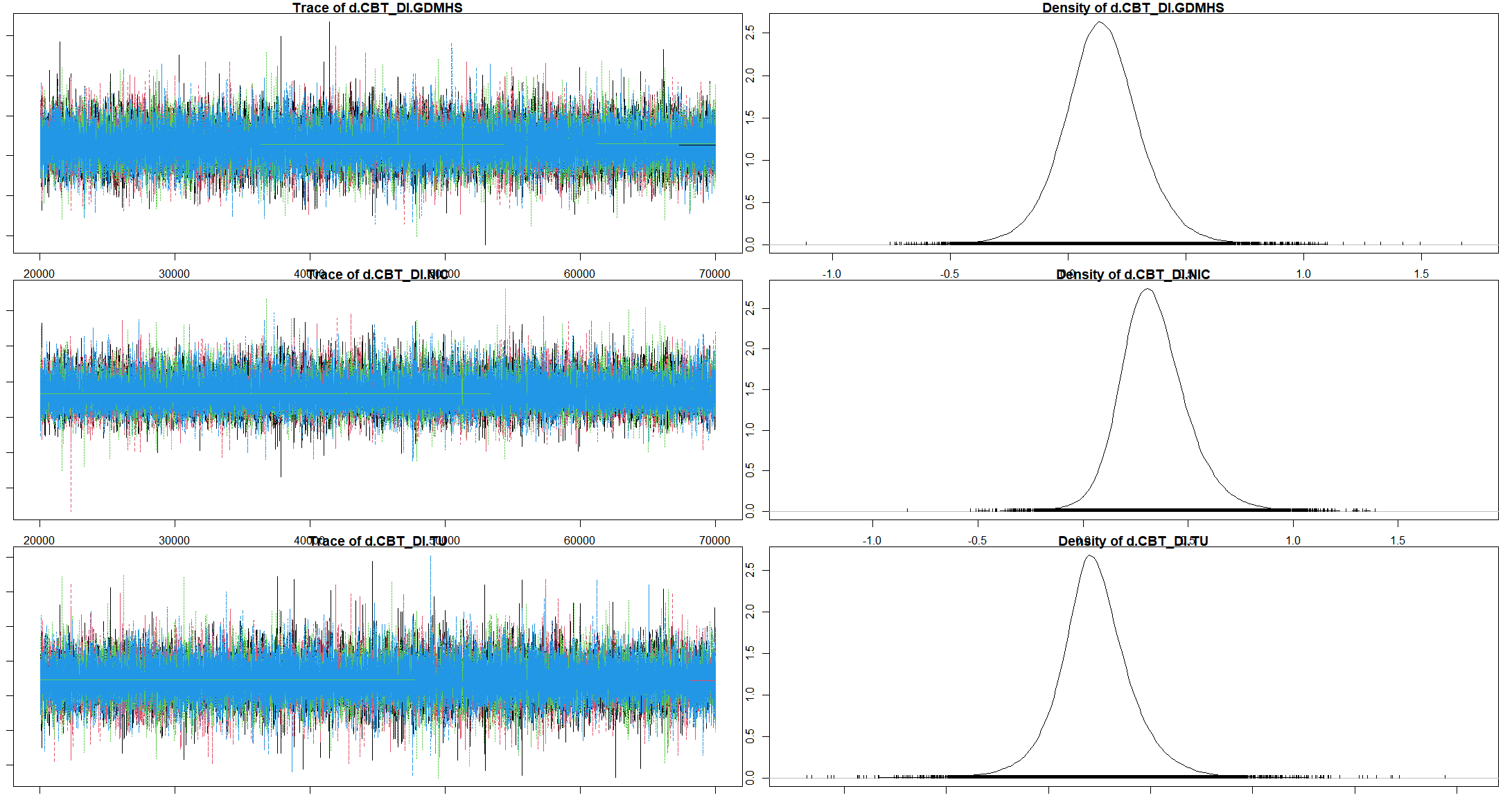 |
| 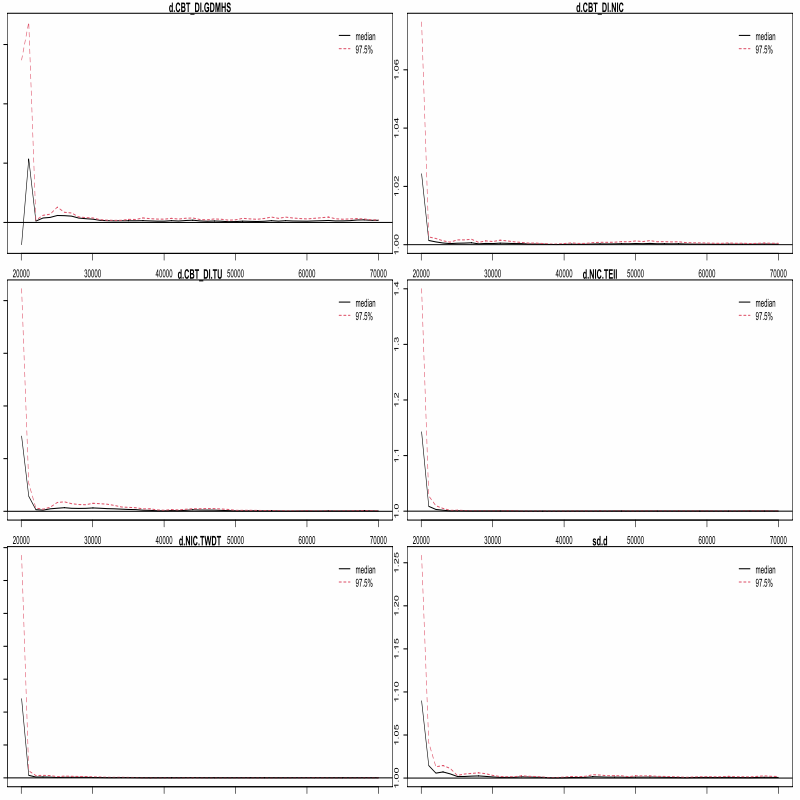 |
| 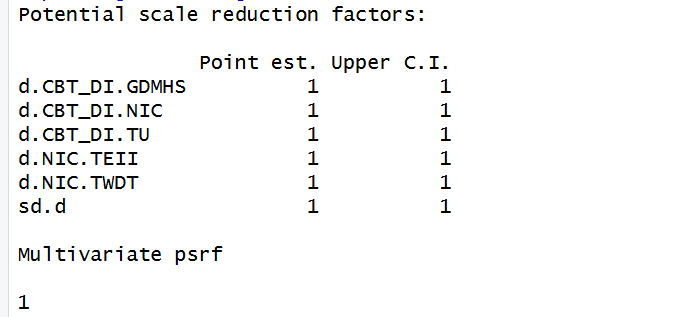 |

Figure S5. MCMC Convergence Diagnostics (Anxiety).

Legend: Convergence diagnostics for the Bayesian model (Anxiety outcomes). (Trace Plots): The trace plots show rapid mixing of the MCMC chains without noticeable trends or divergences, indicating stationarity. (Density Plots): The posterior density plots show well-defined distributions for the model parameters. (Gelman-Rubin Diagnostic): The multivariate PSRF is 1, and individual node PSRF values are all 1.0, providing strong evidence of model convergence.

| 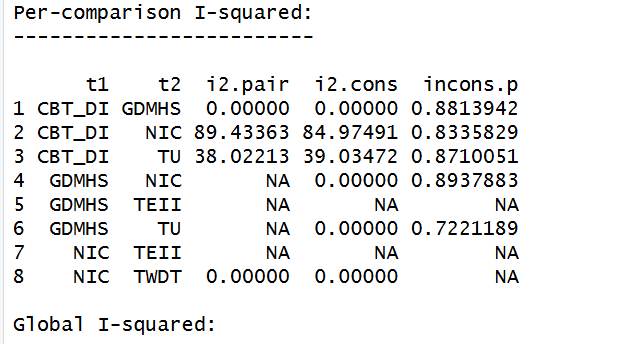 |
| --- |
| 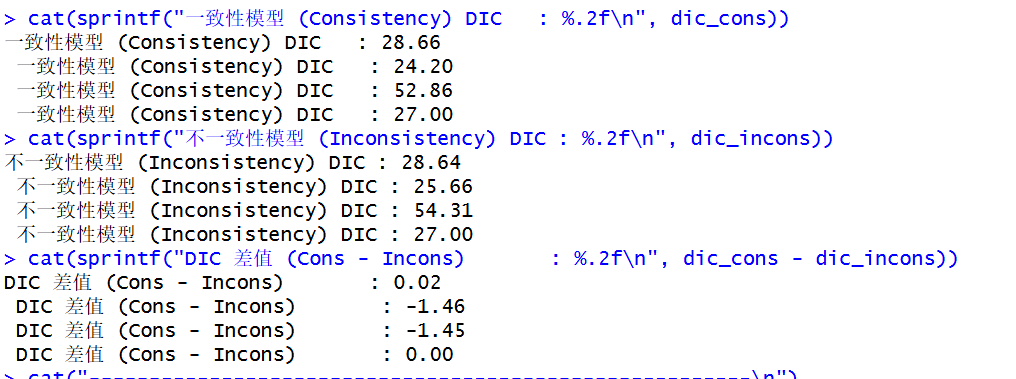 |
| 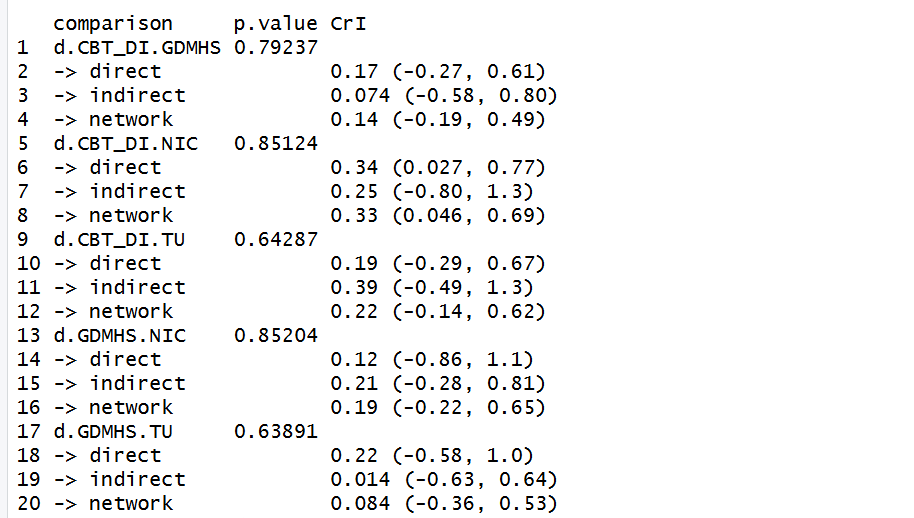 |

Figure S6. Inconsistency Assessment (Anxiety).

Legend: Node-splitting analysis for the anxiety network. This table compares the effect sizes generated from direct comparisons versus indirect comparisons. The P-values for all split nodes are non-significant (> 0.05), suggesting that the direct and indirect evidence sources are consistent and the network model is valid.

| **Depression**  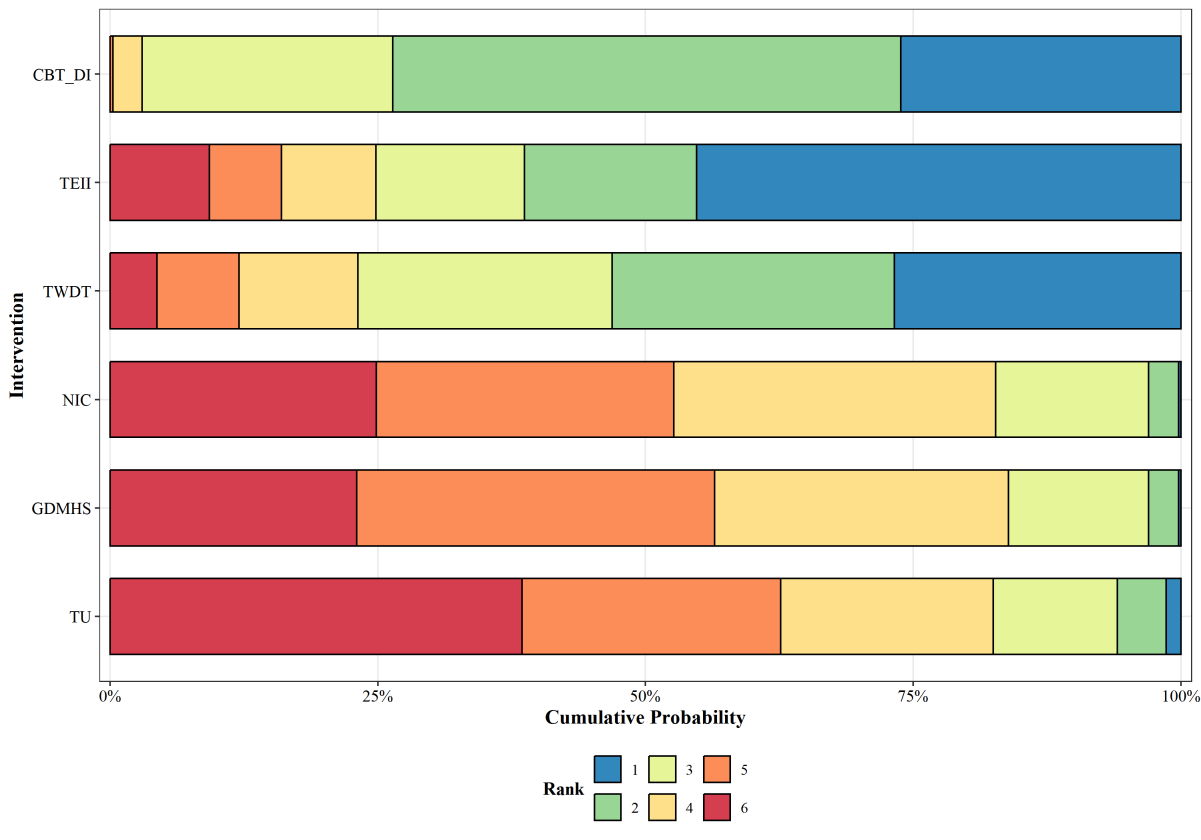 |
| --- |
| 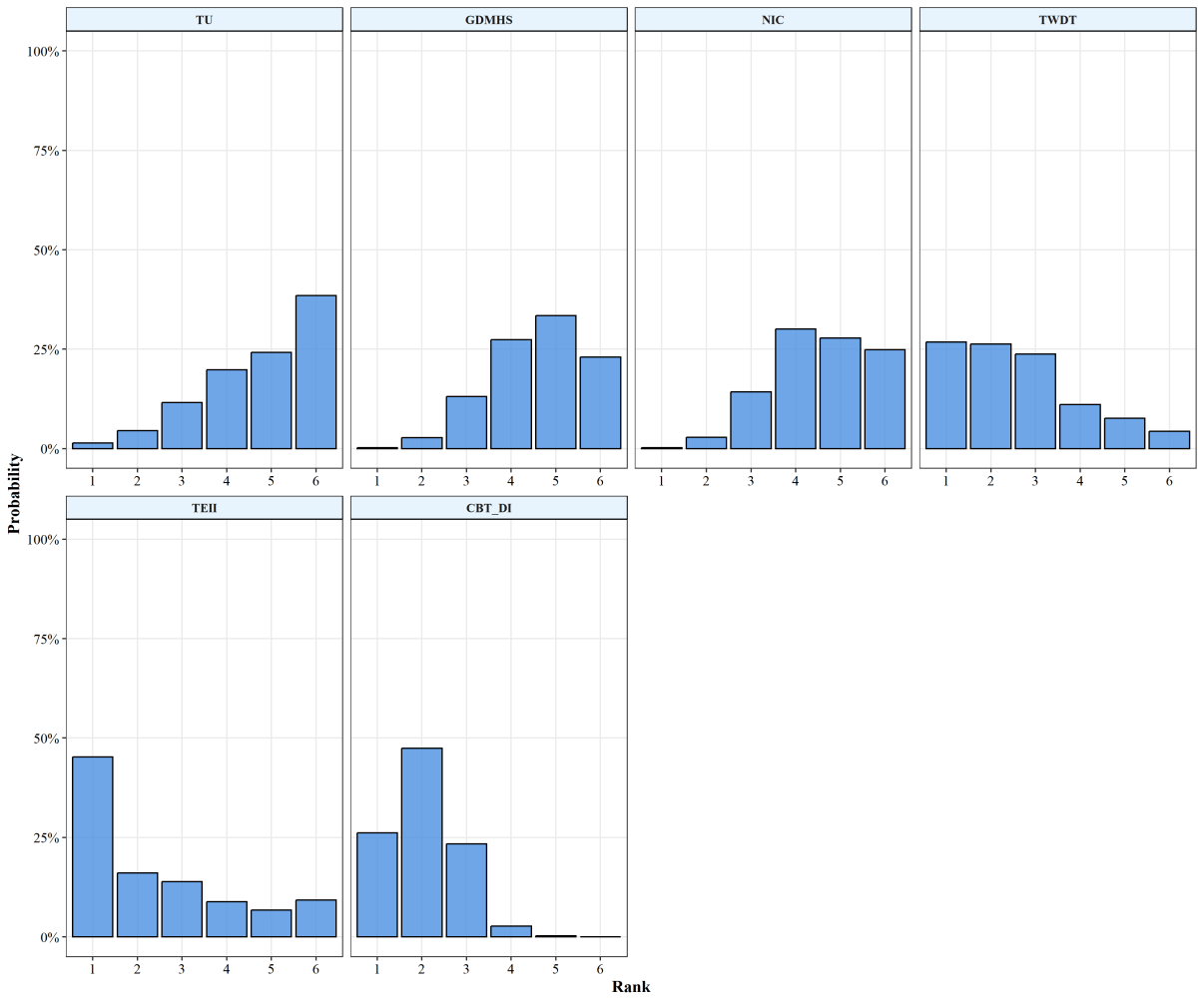 |
| **Anxiety**  **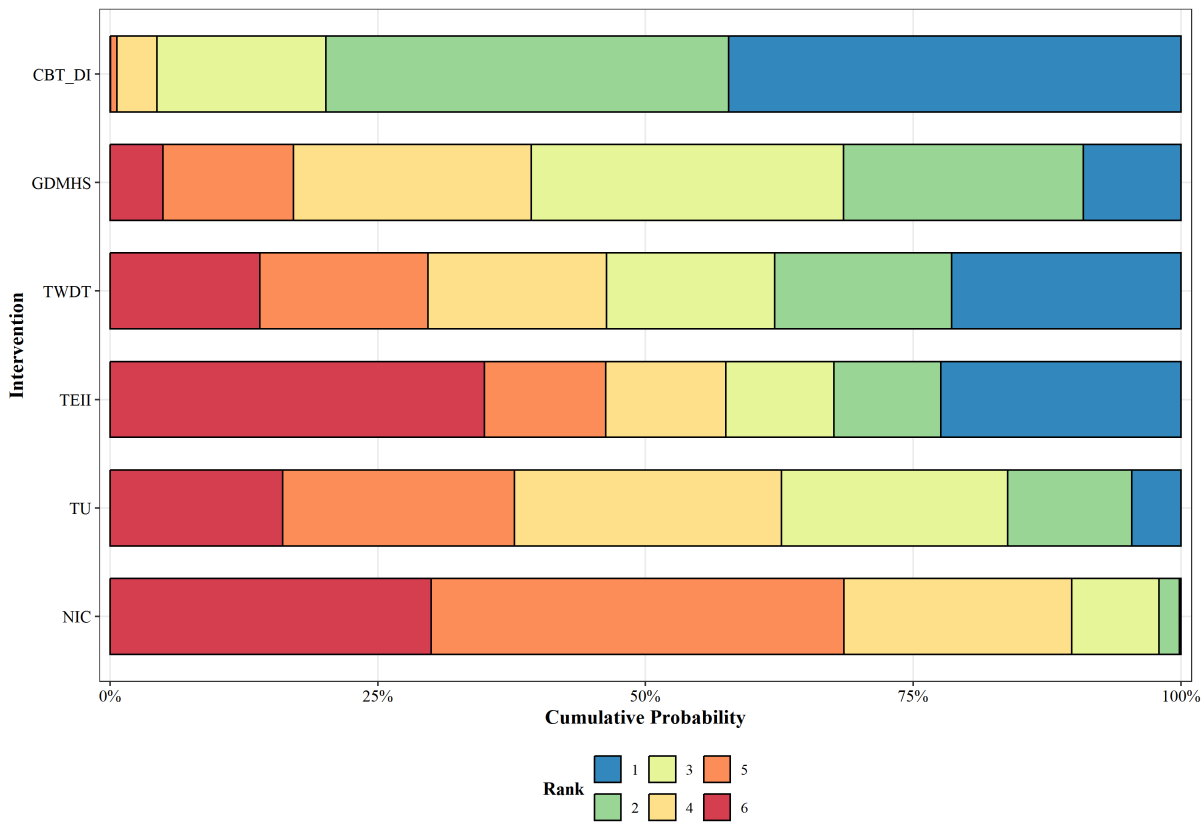** |
| **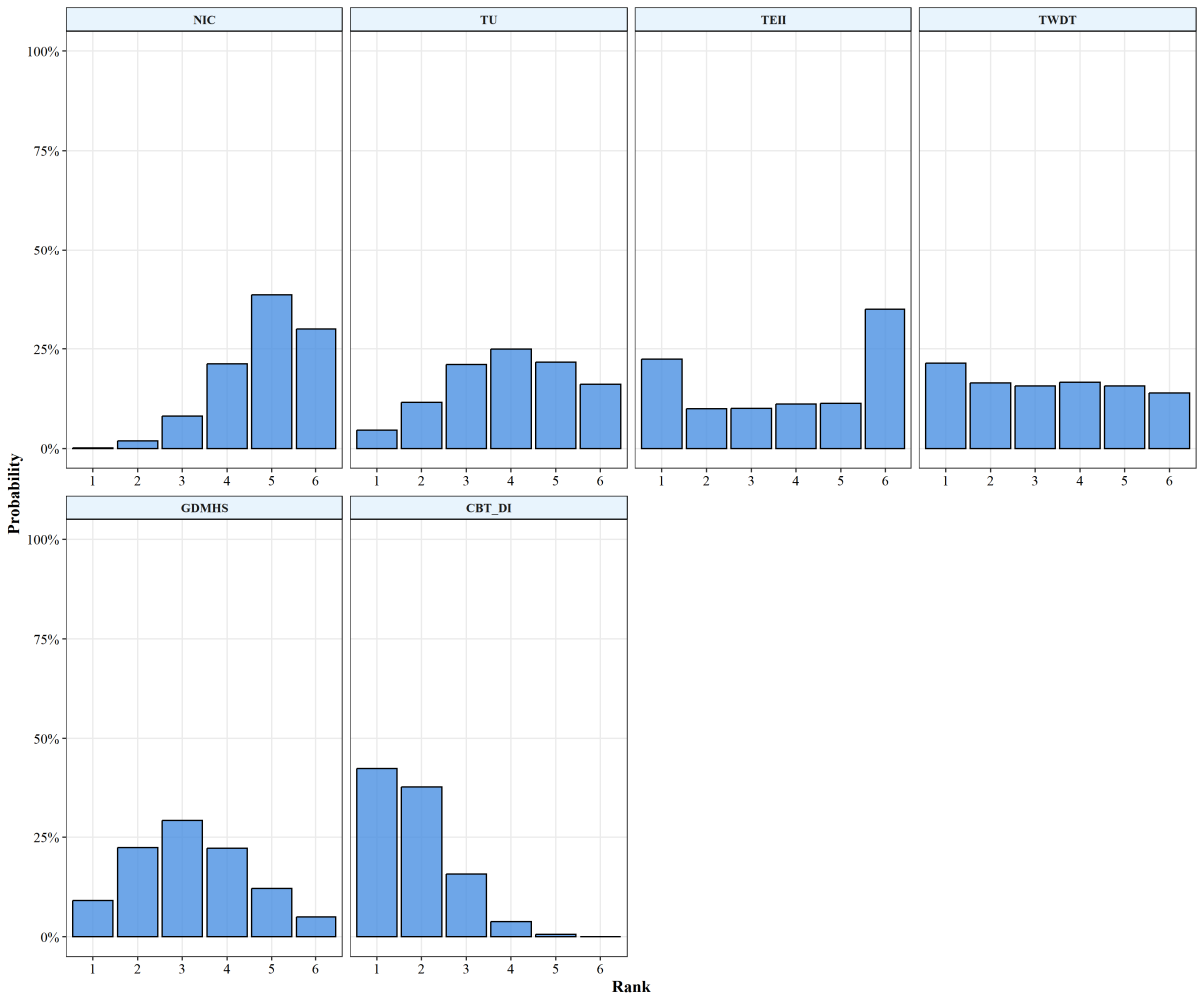** |

Figure S7. Ranking probabilities

Legend: Ranking probabilities for all interventions. (Top Panel) Cumulative Ranking Probability (SUCRA): The horizontal bars represent the cumulative probability of each intervention being ranked among the top n positions. Larger sections of high-rank colors (e.g., Rank 1, 2) indicate a higher probability of being effective. (Bottom Panel) Rankograms: These histograms display the specific probability of each intervention achieving a specific rank (from 1st best to 6th). For example, CBT_DI shows a high probability mass at Rank 1 and Rank 2, indicating it is likely one of the most effective interventions. Abbreviations: CBT_DI: Cognitive Behavioral Therapy-based digital interventions; GDMHS: General Digital Mental Health Support; TEII: Technology-Enhanced Innovative Interventions; TWDT: Third-Wave Digital Therapies; NIC: No Intervention Control; TU: Treatment as Usual.

**Appendix 3: Detailed descriptions of intervention protocols**

| **Study** | **Interventions** | **Comparisongroup(s)** |
| --- | --- | --- |
|  |  |  |
| Barr et al. (2017) | An 8-session computerized cognitive behavioral therapy (CCBT) program, 30-45 minutes each, designed for 12-18 year olds | Accessing currently available self-help websites selected by expert clinical panel, suitable for adolescent age range, with no CBT content |
| Cioffi et al.(2023) | Using Oculus Rift headset for BOXVR boxing game in virtual gym scene, requiring ducking/squatting to avoid obstacles and jabbing/uppercutting to hit targets, 10 minutes per session, 5 times per week for 3 weeks | No participation in any research-related exercise, but completed cognitive tests and anxiety/stress measures at same intervals |
| Deady et al. (2016) | 4-week online self-help intervention based on SHADE program, 1-hour modules per week with homework | 12-module online-attention-control condition where participants read information about various health concerns and complete accompanying surveys |
| Farrer et al. (2024) | 12-module transdiagnostic video intervention, 3-6 minute videos each, featuring animated characters sharing lived experiences of mental health problems, with therapist character presenting psychoeducation and therapeutic techniques | 12 general health topics delivered in PDF format, approximately matched to UVC-Lite on completion time, derived from public domain articles by National Institutes of Health |
| Funk et al. (2025) | An unguided self-help app based on rumination-focused cognitive behavioral therapy (RFCBT) for 6 weeks, designed for 16-22 year olds with elevated levels of RNT | Waitlist control condition offered access to one of the apps after 18 weeks |
| Hallford et al. (2024) | Seven modules of computerized Memory Specificity Training (c-MeST) as adjunct to usual care for 15-25 year olds with MDD | Receiving predominantly psychological therapy or counseling (85%) and/or antidepressants (52%) with no additional intervention |
| He et al. (2022) | An mental health chatbot for 1 week, designed for college students with depressive symptoms | Reading “I Had a Black Dog”, a classic depression book, plus daily high-quality depression-related articles |
| Hoek et al. (2012) | Internet-based guided self-help problem-solving therapy (PST) consisting of 5 weekly lessons. | Waiting list control group (WL) |
| Javadi et al. (2024) | Internet-based Unified Protocol for Transdiagnostic Treatment of Emotional Disorders in Adolescents (UP-A) delivered through video calls on WhatsApp for 2 months | Control group - waiting list group that did not receive treatment |
| Kramer et al. (2014) | Web-based Solution-Focused synchronous chat intervention consisting of individual real-time chat sessions with a trained health care professional in a secured chat room | The waiting list (WL) group did not receive access to the chat intervention. They could participate after the waiting period of 4.5 months |
| Manicavasagar et al. (2014) | Bite Back is an online positive psychology website for adolescents and uses a combination of interactive exercises and information across 9 positive psychology domains: gratitude, optimism, flow, meaning, hope, mindfulness, character strengths, healthy lifestyle, and positive relationships | The two control condition websites that were chosen, Australian Broadcasting Corporation digital channel website, ABC3, and Nine MSN's entertainment website, The Fix, included features that would engage young people and were similar to the Bite Back website |
| Nagamitsu et al. (2022) | Adolescent health promotion interventions using well-care visits and a smartphone cognitive behavioral therapy app. Two intervention groups: (1) WCV group: standardized physical examination along with a structured interview and counseling for youth risk assessment, designed with reference to the Guideline for Health Supervision of Adolescents of Bright Futures; (2) WCV with CBT app group: WCV plus smartphone-based CBT program comprising a 1-week psychoeducation component and a 1-week self-monitoring component | Nonintervention group (control group) - after the intervention period (4 months), participants in the nonintervention group received either WCV or the CBT app (or both) as needed |
| Peake et al. (2024) | A 5-week self-guided cognitive behavioral therapy (CBT) based mobile app intervention, primarily using behavioral activation (BA). It consists of 5 levels (modules) recommended to be completed at a pace of 1 module per week (not expected to take >60 minutes/week). Content includes psychoeducation, mood tracking, activity scheduling, problem-solving, and mindfulness, guided by a therapeutic chatbot character “Limbot”. | An active control app containing 5 modules of age-appropriate psychoeducational content related to the neurobiology of depression (e.g., brain structure, personality). It matched the intervention in duration (5 weeks) and structure (1 module/week) but contained no active CBT or BA components. |
| Stasiak et al. (2014) | The Journey - a computerized cognitive behavioural therapy (cCBT) program for depressed adolescents. Seven modules, each taking approximately 25–30 minutes to complete. Content includes: introduction to CBT model (linking thoughts and actions to feelings), behavioural activation and pleasant activity scheduling, problem solving and conflict resolution, cognitive restructuring (identifying, challenging, and stopping unhelpful thoughts), relaxation techniques, and summary/relapse prevention | Computerized psychoeducation (CPE) program - an attention placebo control program containing simple psycho-educational content including: depression education and mind-body connection, physical health (nutrition, exercise, sleep), friendships and conflict management, time management, stress management, personal fulfillment (creativity, music, poetry), and summary/relapse prevention |
| Teesson et al. (2020) | Climate Schools–Mental Health course, 6 40-min lessons based on cognitive-behavioural principles, aimed at reducing anxiety and depression | Active control - Schools delivered their usual health education classes over the year, including lessons on alcohol, drugs, and mental health |
| Watkins et al. (2024) | The CBT app was based on generic, well established CBT principles and strategies including behavioural activation, problem solving, and spotting and challenging negative thoughts, the CBT app had identical architecture and features and an identical menu to the emotional competence app to match the interventions for delivery, structure, and format, but with different specific CBT content | Personalised emotional competence self-help app based on the component process model of emotion, each participant received content from two of four possible emotional competence modules based on providing two domains of emotional competence ranked worst in an individual’s baseline emotional competence profile |
| Zanden et al. (2012) | The online MYM group course, a structured form of CBT for depression, comprised six sessions of 90 minutes each in a secured chat room | The wait-listed group did not receive an intervention. They were told by email that they would be invited to participate after the waiting period of 3 months |
| Zhou et al. (2023) | A automated self-help app based on Stress Inoculation Training (SIT), a type of Cognitive Behavioural Therapy aimed at reducing stress, consisting of 11 sessions delivered over 11 weeks with each session lasting between 25–45 minutes. The modified SIT maintained the same phases as standard SIT, with the skill training phase including somatic skills (progressive relaxation and mindfulness), cognitive restructuring skills, behavioural skills (goal setting, time management, and problem-solving skills), and interpersonal skills | Control group students attended their regular moral education classes as arranged by the schools, which typically included non-academic activities such as watching news or head teachers discussing daily class affairs |

Note: This table summarizes the specific intervention content, delivery format, duration, and control conditions for each included study.

**Appendix 4: Sensitivity Analysis And Publication Bias**


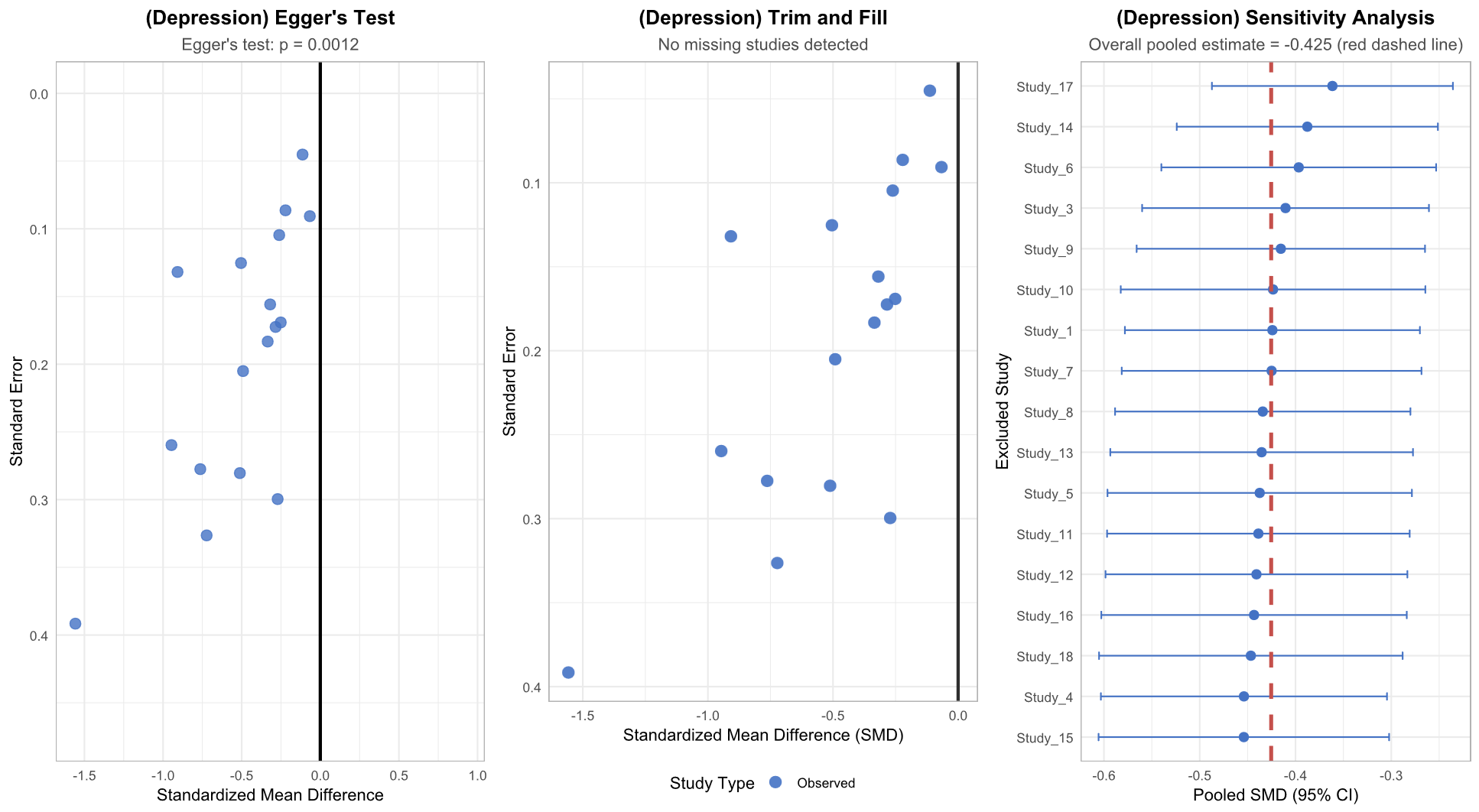


Figure S8. Assessment of Publication Bias and Sensitivity Analysis for Depression Outcomes.

Legend: This composite figure displays the robustness checks for depressive symptoms. (Left Panel) Egger’s Linear Regression Test: The scatter plot visualizes the relationship between the standardized mean difference (SMD) and standard error. The vertical line represents the summary effect size. The significant P-value (P = 0.0012) indicates asymmetry in the funnel plot, suggesting potential publication bias or small-study effects. (Middle Panel) Trim and Fill Analysis: This method attempts to correct for funnel plot asymmetry. The blue dots represent observed studies. No yellow triangles (imputed missing studies) were detected, suggesting that despite the asymmetry, the method did not find evidence of missing studies to adjust the effect size. (Right Panel) Leave-One-Out Sensitivity Analysis: This forest plot assesses the stability of the pooled result. The red vertical dashed line represents the original pooled SMD (-0.425) including all studies. Each blue point represents the recalculated pooled SMD (with 95% CI) after excluding the specific study listed on the y-axis. The results show that the effect size remains stable and does not cross zero or change direction regardless of which study is removed.


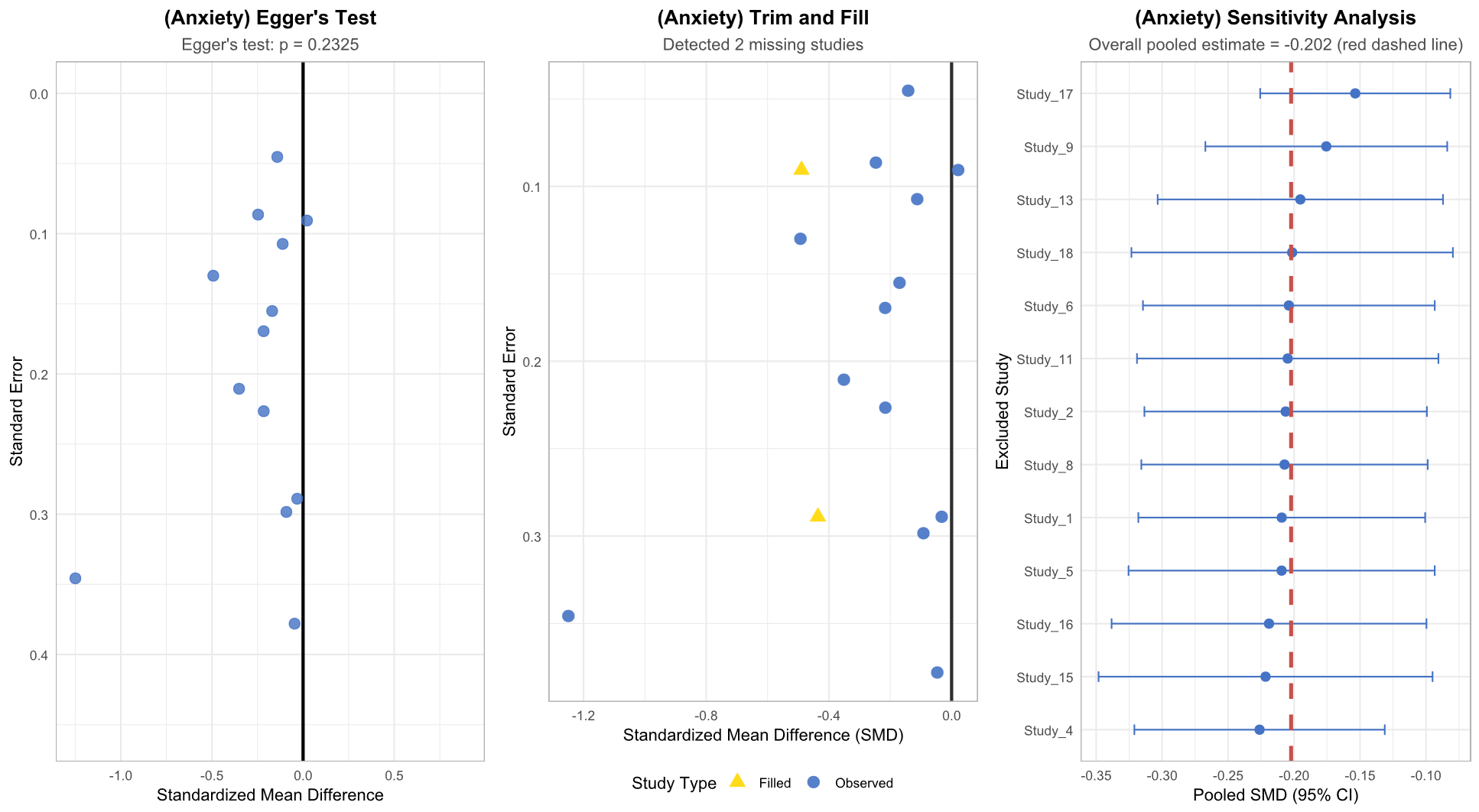


Figure S9. Assessment of Publication Bias and Sensitivity Analysis for Anxiety Outcomes.

Legend: This composite figure displays the robustness checks for anxiety symptoms. (Left Panel) Egger’s Linear Regression Test: The P-value (P = 0.2325) is greater than 0.05, indicating no statistically significant funnel plot asymmetry and a lower risk of publication bias compared to depression outcomes. (Middle Panel) Trim and Fill Analysis: The method identified 2 potential missing studies (indicated by yellow triangles) to correct for minor asymmetry. However, the adjustment (shifting from observed blue dots to the corrected estimate) did not fundamentally alter the direction of the treatment effect. (Right Panel) Leave-One-Out Sensitivity Analysis: The red vertical dashed line indicates the original overall pooled estimate (-0.202). The plot demonstrates high robustness, as omitting any single study resulted in pooled estimates that fluctuated only slightly and consistently overlapped with the original confidence interval.
